# A genomic perspective on the important genetic mechanisms of upland adaptation of rice

**DOI:** 10.1186/1471-2229-14-160

**Published:** 2014-06-11

**Authors:** Jun Lyu, Baoye Li, Weiming He, Shilai Zhang, Zhiheng Gou, Jing Zhang, Liyun Meng, Xin Li, Dayun Tao, Wangqi Huang, Fengyi Hu, Wen Wang

**Affiliations:** 1 CAS-Max Planck Junior Research Group, State Key Laboratory of Genetic Resources and Evolution, Kunming Institute of Zoology, Chinese Academy of Sciences, Kunming 650223, China; 2 Food Crops Research Institute, Yunnan Academy of Agricultural Sciences, Kunming 650205, China; 3 BGI-Shenzhen, Shenzhen 518083, China; 4 Inner Mongolia Agricultural University, Hohhot 010018, China; 5 Center for Epigenetics, Johns Hopkins University School of Medicine Baltimore, MD, Baltimore 21205, USA

**Keywords:** Upland rice, Upland adaptation, Genetic mechanisms, Phylogenetics, Population structure, Artificial selection

## Abstract

**Background:**

Cultivated rice consists of two important ecotypes, upland and irrigated, that have respectively adapted to either dry land or irrigated cultivation. Upland rice, widely adopted in rainfed upland areas in virtue of its little water requirement, contains abundant untapped genetic resources, such as genes for drought adaptation. With water shortage exacerbated and population expanding, the need for breeding crop varieties with drought adaptation becomes more and more urgent. However, a previous oversight in upland rice research reveals little information regarding its genetic mechanisms for upland adaption, greatly hindering progress in harnessing its genetic resources for breeding and cultivation.

**Results:**

In this study, we selected 84 upland and 82 irrigated accessions from all over the world, phenotyped them under both irrigated and dry land environments, and investigated the phylogenetic relations and population structure of the upland ecotype using whole genome variation data. Further comparative analysis yields a list of differentiated genes that may account for the phenotypic and physiological differences between upland and irrigated rice.

**Conclusions:**

This study represents the first genomic investigation in a large sample of upland rice, providing valuable gene list for understanding upland rice adaptation, especially drought-related adaptation, and its subsequent utilization in modern agriculture.

## Background

Asian cultivated rice (*Oryza sativa* type indica and *Oryza sativa*. type japonica) has phenotypically divergent ecotypes [1]. Irrigated and upland rice, respectively adapted to growing in paddy fields and rain-fed dry lands, are two main ecotypes that are generally considered to show apparent phenotypic differentiation. The upland type is more drought tolerant, taller, of lower tillering potential, more resistant to blast, and tends to have longer and thicker roots than its irrigated counterpart [2,3]. Accordingly, upland varieties are widely adopted in upland areas to optimize crop yields because of their more efficient water usage and adaptation to dry land. Though the yield of upland rice is comparatively lower than the irrigated rice, it provides the daily staple food on which nearly one hundred million people depends [4]. Because we are now facing global population expansion and a growing shortage of water resources, upland rice and especially knowledge about its genetic mechanisms for dry land adaptation become more important as they allow the possibility of transforming or modifying high-yield irrigated varieties of rice to upland ones.

Drought resistance is an important component of rice adaption to most rainfed upland areas because in this ecosystem water could not be well preserved and frequent or sporadic drought events could occur each year [5]. Previous research proposed three different mechanisms for plant drought resistance: drought escape, dehydration avoidance, and dehydration tolerance [6]. Through drought escape, timing of plant lifecycle is modulated to escape the time of drought when drought time is predictable in each year. Dehydration avoidance refers to the case that plants constitutively avoid excess loss of water through more developed root systems or a thick layer of cutin [6]. Dehydration tolerance refers to the inducible mechanisms, like osmotic adjustment, which help plants to prevent damage and survive when excess loss of water does happen [6]. For the frequent and unpredictable drought stress in upland areas, the dehydration avoidance and dehydration tolerance should be the appropriate options for upland crops. In fact, upland rice seems to depend more on the constitutive dehydration avoidance than the inducible dehydration tolerance in its adaptation [7-9]. By far, some genes have been reported to be related with drought resistance[10], such as genes in abscisic acid (ABA) synthetic pathway (such as 9-*cis*-epoxycarotenoid dioxygenase genes) [11,12], transcription factors interacting with the cis-acting dehydration response element (DRE) [13], genes synthesizing osmoprotectants [14,15], and mitogen-activated protein kinase (MAPK) genes [16]. However, these studies focused on single gene’s effect, while adaptation to dry land may involve many genes in the genome. Furthermore, the valuable genetic resources within upland rice, which were ignored by most previous studies, are also worthy of enhanced utilization.

Upland and irrigated rice evolve their respective characteristic traits under both natural and artificial selections, which lead to phenotypic adaptation to respective environment and simultaneously genetic differentiation between ecotypes. Some statistical tools for identifying selected regions during population differentiation have been well established. Fixation index (*F*_ST_) is a commonly used statistics that measure population differentiation based on genetic polymorphism data. *F*_ST_ is positively associated with allele frequency difference. Cross-population composite likelihood ratio test (XP-CLR), a newly invented approach, incorporates not only genetic differentiation but also allele frequency spectrum information [17]. By jointly modeling multilocus allele frequency differentiation and frequency spectrum under neutrality and selection, XP-CLR was demonstrated to have high power in detecting selective sweeps during population differentiation. Besides, Nielsen’s CLR, a parametric test also based on composite likelihood ratio, detects selective sweeps using the spatial distribution of site frequency spectrum [18]. This approach only uses SNP data of one population and thus detects selective sweeps in single population without consideration of population differentiation.

To investigate the genetic mechanisms for upland adaptation and identify genes for its important component, ‘drought resistance’, we use our whole-genome re-sequencing data for a large panel of accessions, including 84 upland accessions and 82 irrigated accessions reported by our previous work [19] , where these data were used to test that a low-diversity region containing a key enzyme gene of abscisic acid synthesis was due to human selection. Considering the wide distribution of upland rice in the world, we obtained our samples from Asia, Africa and South America, covering most major distribution areas of upland rice. We explored phylogenetic relationship between upland and irrigated ecotypes and population structure of current upland rice population. More importantly, by detecting selective sweep regions using *F*_ST_ and XP-CLR, we compiled a list of candidate genes that are likely responsible for upland adaptation or that are related to phenotypic differentiation between the upland and irrigated ecotypes, providing valuable insights about crop breeding for dry land.

## Results

### Phenotypes of the upland accessions differ from those of the irrigated ones

For the 84 globally sampled and representative upland rice accessions (see Additional files 1 and 2), we want to confirm whether they possess the reported phenotypes that distinguish them from irrigated rice [2,3]. Therefore we grew the varieties to do phenotyping (see Additional files 3 and 4). Our examination focused on four non-yield phenotypes that are reported to be differentiated between upland and irrigated rice: root weight, maximal main root length, tiller number, and plant height. Moreover, we also examined four yield traits, including thousand grain (1000-grain) weight, number of productive panicles, number of filled grain per panicle and number of empty grain per panicle. Under both well-watering and dry land (rainfed upland) environments, the upland accessions consistently showed significantly taller plant architecture, fewer tillers, longer main roots and heavier root weights, which supports the previously reported phenotypic differences (Table 1, see Additional file 5 for the phenotypic difference). Regarding the yield traits, under both environments upland accessions show larger 1000-grain weight and less productive panicles as compared to irrigated accessions (Table 1). The numbers of filled and empty grain per panicle show no significant differences between the two ecotypes under the irrigated condition. However, under the rainfed upland condition, the upland accessions perform much better, i.e. have significantly more filled grain (Student’s t-test, *P* = 0.016) and less empty grain (Student’s t-test, *P* = 5.3e-05) per panicle than the irrigated accessions.

**Table 1:** Phenotypic differentiation between upland and irrigated rice

**Environment**	Phenotypes*	**Irrigated rice**	**Upland rice**	***P*-value**	**Mean difference**
Between ecotypes/percentage					
Irrigated environment	Plant height (cm)	89.5 ± 16.0	116.1 ± 22.1	6.5e-12	26.6/29.7%
	Tillering number	9.8 ± 3.1	8.7 ± 2.7	0.042	1.1/11.2%
	Root weight (g)	12.8 ± 5.7	17.2 ± 7.1	0.00029	4.4 /34.4%
	Maximal main root length (cm)	24.1 ± 2.5	27.2 ± 4.1	2.0e-06	3.1/12.9%
	Thousand grain weight (g)	23.8 ± 2.9	25.9 ± 4.3	0.0020	2.1/8.8%
	Number of productive panicles^@^	11.0 ± 3.1	9.6 ± 2.7	0.015	1.4/12.7%
	Number of filled grain per panicle	124.2 ± 27.6	129.8 ± 35.3	0.33	5.6/4.5%
	Number of empty grain per panicle	31.0 ± 21.1	31.4 ± 15.4	0.91	0.4/1.3%
Upland environment	Plant height (cm)	81.2 ± 12.6	103.9 ± 15.4	7.3e-16	22.7/28.0%
	Tillering number (∆ number of productive panicles)	1.5 ± 0.6	1.3 ± 0.5	0.024	0.2/13.3%
	Root weight (g)	1.8 ± 0.9	2.4 ± 1.0	0.00031	0.6/33.3%
	Maximal main root length (cm)	23.5 ± 4.0	29.9 ± 6.1	6.9e-11	6.4/27.2%
	Thousand grain weight (g)	23.4 ± 4.7	25.6 ± 4.4	0.0071	2.2/9.4%
	Number of filled grain per panicle	33.3 ± 16.6	40.5 ± 16.9	0.016	7.2/21.6%
	Number of empty grain per panicle	26.3 ± 10.1	18.9 ± 9.8	5.3e-05	7.4/28.1%
Mean differences between environments #	Plant height (cm)/percentage	-8.3/-9.3%	-12.2/-10.5%		
	Tillering number/percentage	-8.3/84.7%	-7.4/-85.1%		
			-14.8/-86.0%		
	Root weight (g)/percentage		+2.7/+9.9%		
		-11.0/-85.9%			
	Maximal main root length (cm)/percentage				
		-0.6/-2.5%			
	Thousand grain weight	-0.4/-1.7%	-0.3/-1.2%		
	Number of productive panicles	-9.5/-86.4%	-7.41/-85.0%		
	Number of filled grain per panicle	-90.9/-73.2%	-89.3/-68.8%		
	Number of empty grain per panicle	-4.7/-15.2%	-12.5/-39.8%		

*Phenotypic value is indicated by mean value ± standard deviation.

#The difference is positive when the phenotypic value in the upland environment is larger than in the irrigated environment, otherwise the difference is negative.

^@^The number of productive panicles were measured at the stage of maturity, while tiller number was assessed before the earing period. Since different accessions have different heading dates, some accessions produce more tillers after we measured the tiller numbers. So the final mean numbers of productive panicles were larger than the mean tiller numbers in the irrigated condition.

∆ In the upland environment, most of the accessions have less than two tillers. When we phenotyped the number of productive panicles, we found almost all the tillers develop productive panicles, i.e. the percentage of earbearing tiller is close to 100% in the rainfed upland condition. So here in the upland environment, tiller numbers and productive panicle numbers share the same values. The tillering ability is weak in our upland condition, and no more tillers were produced at the stage of maturity.

We focus on four non-yield phenotypes in this study, including plant height, tillering number, root weight and maximal main root length, and four yield phenotypes, including thousand grain weight, number of productive panicles, number of filled grain per panicle and number of empty grain per panicle. These phenotypic data were collected under both irrigated and upland environments.

Using software SPSS statistics 17.0, we calculated the pearson correlation coefficients (ρ) between each of the four non-yield traits and each of the yield traits under upland condition (Additional file 6). The plant height is significantly associated with the 1000-grain weight (ρ = 0.263, *P* = 0.002), the number of empty grain per panicle (ρ = −0.226, *P* = 0.009) and the number of productive panicles (ρ = −0.2, *P* = 0.022). The tiller number is significantly associated with the number of productive panicles (ρ ≈ 1, *P* < 0.001) and the 1000-grain weight (ρ = −0.169, *P* = 0.05). The root weight is significantly associated with the number of filled grain per panicle (ρ = 0.295, *P* = 0.001). The maximal root length is significantly associated with the thousand grain weight (ρ = 0.443, *P* < 0.001) and the number of empty grain per panicle (ρ = −0.232, *P* = 0.008). Therefore all of the four non-yield traits are significantly associated with yield traits under rainfed upland condition. From the ρ values it could be speculated that larger root weight and greater root length of upland rice would contribute to net yield increase under upland condition probably because better developed root systems could contribute to better dehydration avoidance. However, the effects of plant height and tiller number on yield are complex. The larger plant height of upland accessions would possibly cause the reduction of productive panicles, but can increase 1000-grain weight and reduce the number of empty grain. The less tiller numbers of upland accessions can cause reduction of productive panicles, but would increase 1000-grain weight.

When comparing these phenotypes between the two culture conditions, we found that dry land environment can slightly reduce plant height and 1000-grain weight, but will dramatically decrease the tiller number, root weight, number of productive panicles and number of filled grain per panicle of both the upland and the irrigated ecotypes. However, the main root length and the number of empty grain per panicle responded differently to the different environments between the two ecotypes. We observed a minor decrease (−2.5%) of main root length of irrigated rice but a significant increase (+9.9%) of upland rice root length in dry land as compared with that in the irrigated condition (Table 1, Student’s t-test, *P* = 0.002, Additional file 7), indicating an adaptive feature of upland rice. Specifically, 61 percent of the upland accessions showed increase of root length in the upland condition, while only about 38 percent of the irrigated accessions showed root length increase in the upland condition. Moreover, for the number of empty grain per panicle, we observed a slight and nonsignificant reduction of it for the irrigated accessions in the upland condition (Student’s t-test, *P* = 0.29, Additional file 7) but a dramatic decrease of it for the upland accessions (Student’s t-test, *P* = 5.9e-07, Additional file 7), suggesting another adaptive trait of upland rice. Based on the phenotypic differentiation between the upland and irrigated accessions and the correlations between the non-yield phenotypes and yield phenotypes in the upland condition, we define ‘upland adaptation’ in this study as the characteristic non-yield phenotypes of upland rice that are associated with the yield performance in upland conditions, such as plant height, tiller number, root weight and maximal root length which are directly associated with drought resistance.

### SNPs calling and annotation for the 166 accessions

For the 82 irrigated and 84 upland accessions, we obtained 402,052,644 pair-end reads that passed the reads Pass Filter, amounting to around 8 G base pairs (NCBI accession code SRA066116). The re-sequencing data for 84 upland and 82 irrigated accessions was reported in our previous work [19]. Here we comprehensively analyze these re-sequencing data to determine the genetic components that might have caused the upland adaptation and phenotypic differentiation between the two ecotypes, which has been a long-term open question in rice study.

About 87 percent of these reads could be aligned to the reference genome (IRGSP/RAP build 5 Nipponbare genome) using SOAP-2.20 software [20]. The average mapping ratios of reads for the type indica and type japonica are 81% and 92%, respectively. On average, 29.54% of the reference genome was covered for each accession (ranging from 18.65% to 46.58%). The mean depth for each accession was about 0.50 × on average (ranging from 0.27 × to 0.96 ×) (see Additional file 8). In total, the genome coverage of the upland and irrigated population reached 41 × and 43 ×, respectively. After SNP calling and a series of filtering for quality control (see Methods), 3,571,104 high-quality SNPs were identified (Additional file 9 includes all the SNPs), of which 3,563,681 are non-singleton SNPs. By randomly choosing 56 SNPs and genotyping them via Sanger sequencing, we were able to validate our SNP calling accuracy at 96.4%. The sequencing data, though relatively low in depth, are enough to well satisfy our analyses, as we shall discuss below.

In the following analyses, only 3,029,822 SNPs with genotype data covering more than 10 individuals in both populations were used. We annotated these SNPs by classifying them into different categories according to the genomic context (see Additional file 10). The non-synonymous-to-synonymous ratio of SNPs is 1.32, consistent with the previously reported ratio of 1.29 in rice [21]. SNPs causing radical mutations, such as premature stop codons or frame disruptions, were also identified (Additional file 10).

### Phylogenetics and population genetics analysis for upland rice

**Figure 1:**
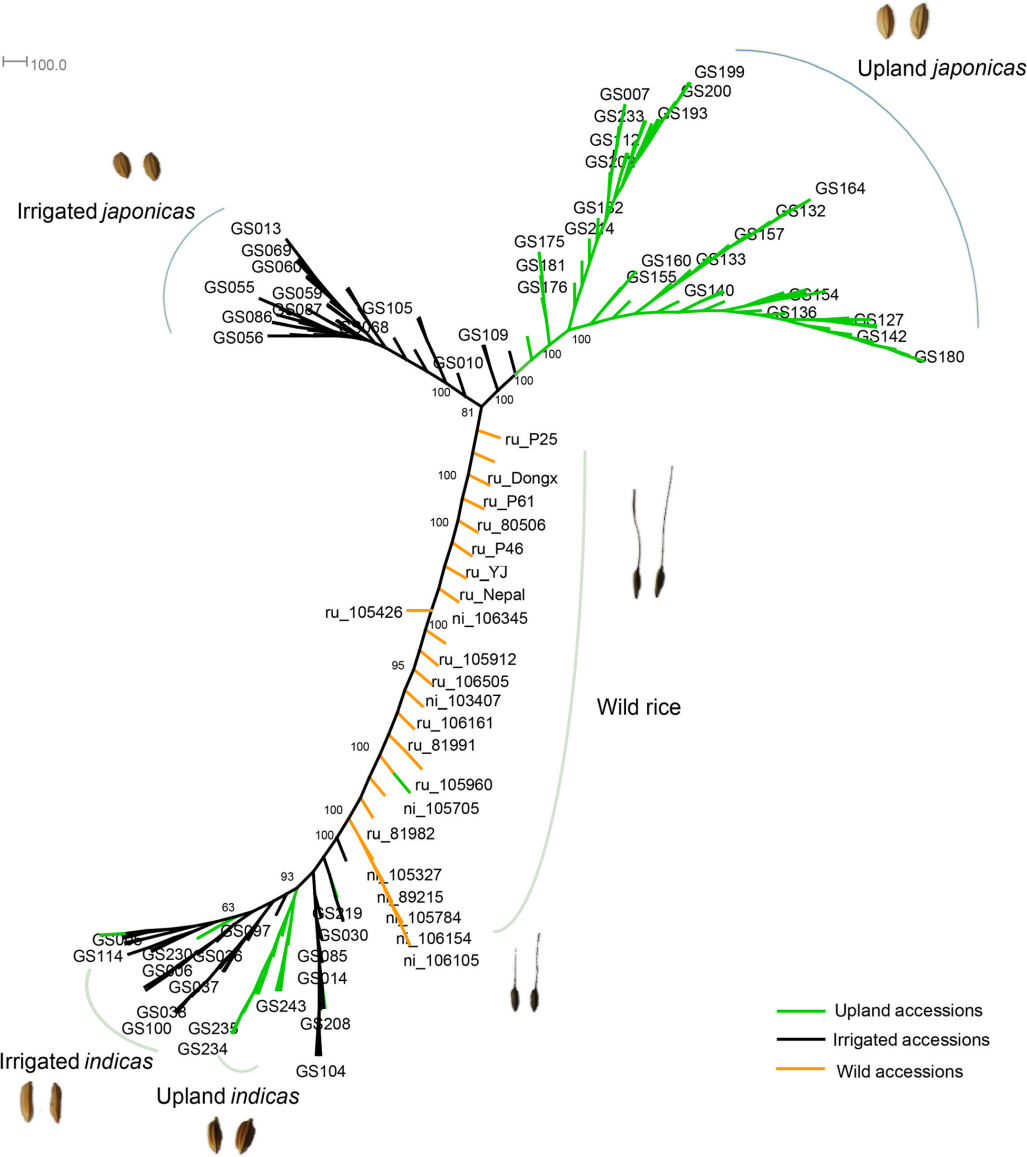
**Phylogenetic tree of rice accessions.** Green, black and orange branches refer to upland, irrigated and wild accessions respectively. Analysis showed that differentiation between Indica and Japonica has existed within the wild population, since there are strains of wild rice close to both Indica and Japonica respectively, supporting the double domestication model. The tree shows multiple origins for upland rice, though the upland japonicas may bear a single origin. Bootstrap values are indicated in some of the major internal nodes. Some of the leaf nodes are labeled with the sample number of the rice accessions. ‘ru’ refers to ‘*rufipogon*’, and ‘ni’ refers to ‘*nivara*’.

SNP genotypes were used in phylogenetic analysis for the 166 accessions. Additionally, 25 wild accessions from our previous data (10 *O. nivara* and 15 *O. rufipogon*, Additional file 11) were added to help address the phylogenetics of upland accessions [21]. We constructed the accessions’ phylogenetic tree using a Neighbor-Joining approach with 100 bootstraps (Figure 1). Consistent with some of the previous reports [21], our results support that Indica and Japonica usually considered as two subspecies are very different groups, as they largely clustered in respective group and both have closely related wild accessions (Figure 1, also see Additional file 12). The results also show that probably the majority of current upland accessions belong to the type japonica (in our sample 76% upland accessions are japonica) (Figure 1, Additional file 12), consistent with the classification of the collection center based on traditional phenotypic characteristics and previous reports that upland rice mainly pertains to Japonica [22]. The phylogenetic tree showed that all the upland japonica accessions cluster together, suggesting that upland japonica likely have a single origin. However, for the type indica, the scattering pattern of upland indica indicates that upland indica accessions may have been separately bred multiple times using different irrigated rice as the basic genetic materials.

### Population structure in upland rice and admixture in upland indica

**Figure 2:**
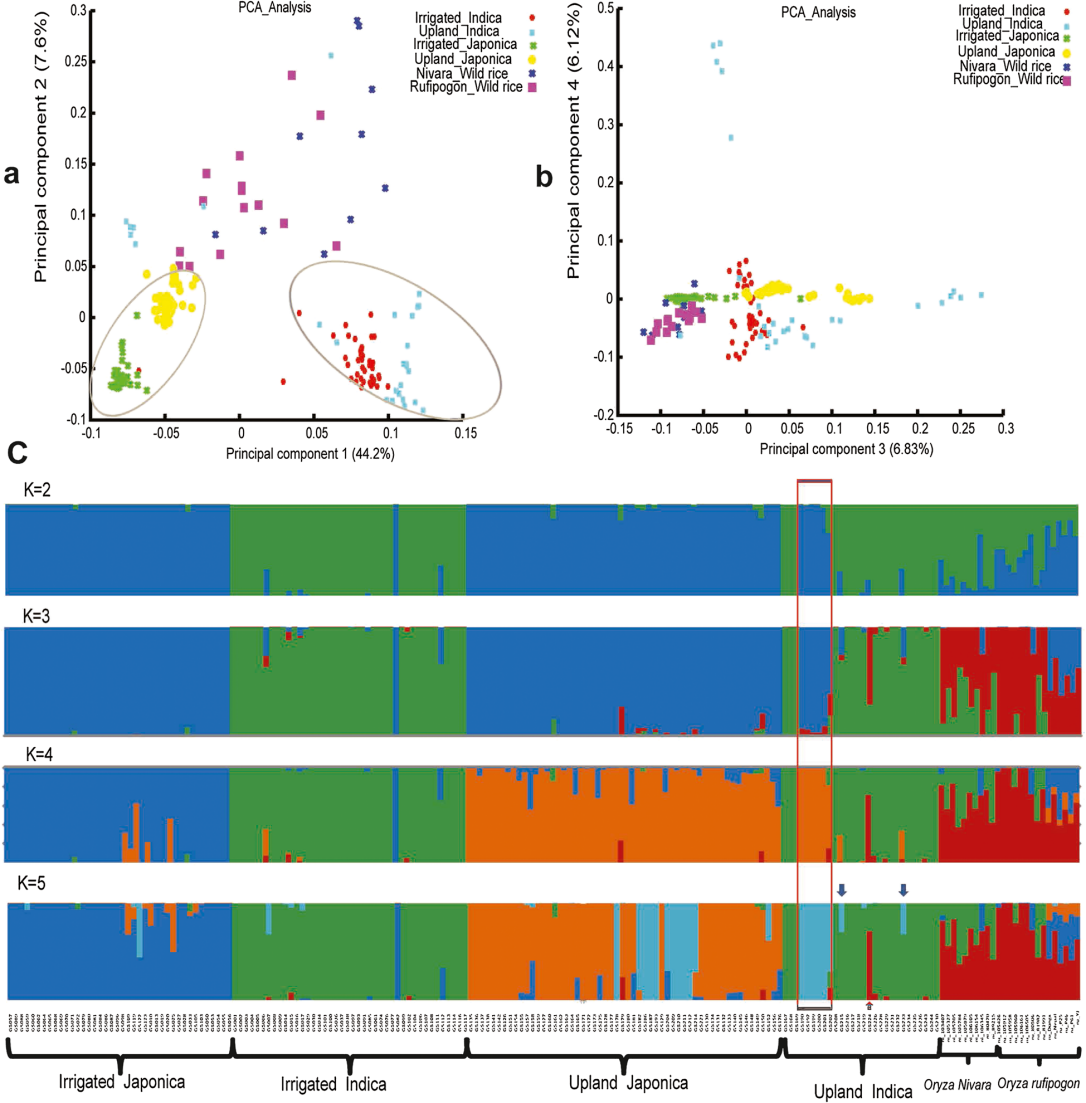
**Population structure estimation.** PCA analysis was conducted with the top four components shown in **(a)** and **(b)**. The four components together explain about 65% of the genetic variance. **(a)**, Principal component 1 distinguishes the type japonica from the type indica. Both types include two ecotypes (upland and irrigated ones). The ecotype differentiation in Japonica is clear-cut. **(b)**, principal components 3 and 4. **(c)**, the population structure reported by FRAPPE. Results were shown from K = 2 to K = 5. When K = 2, there is only division of Indica and Japonica. Then wild rice is separated when K = 3. When K = 4, the ecotype differentiation in Japonica emerges, i.e. upland japonica begins to differ from irrigated japonica. Our results show that upland-irrigated ecotype differentiation is genetically lower than the Indica-Japonica differentiation. The red rectangle indicates the six upland accessions, which were previously classified as upland indica accessions but are genetically more close to the upland japonica group. The blue arrows in K = 5 panel indicates the two upland indica, GS215 and GS233, which have a large genomic proportion close to upland japonica. The red arrow indicates upland indica, GS224, which is close to wild rice.

We use 24,890 SNPs with genotype data covering more than 100 accessions to analyze the population structure within our samples (see Methods). Principle component analysis (PCA) summarizes the population structure for the entire population (Figure 2a, b). The top four principle components explain about 65% of the genetic variation. The huge differentiation on principle component 1 distinguishes Indica from Japonica. By contrast, genetic differentiations between upland and irrigated ecotypes seem to be much lower. The *F*_ST_ value for the two ecotypes calculated based on genome-wide SNPs is only 0.06. Even in the type japonica, where the upland accessions approximate monophyly and the ecotype differentiation is clear-cut (Figure 1, Figure 2a), the upland and irrigated ecotypes still have a limited genetic differentiation (*F*_ST_ = 0.13), much lower than the reported differentiation between type indica and type japonica (*F*_ST_ = 0.55) [23].

We also investigated the population structure using FRAPPE to estimate the ancestry and population admixture of each accessions [24]. By increasing K (the number of populations) from 2 to 5, the population structure of upland and irrigated accessions are shown (Figure 2c). For K = 2, we see a differentiation between type indica and type japonica, and this differentiation also exists for *Oryza rufipogon* and *Oryza nivara* in the wild population. When K = 3, the wild rice and the cultivars become separated. When K = 4, the division between irrigated japonica and upland japonica emerge, while the irrigated indica and upland indica still share the same structure, indicating the ecotype differentiation is higher within the type japonica than in the type indica. When K = 5, a new subgroup in upland japonica appears, suggesting some accessions in upland japonica forms a unique subgroup which is differentiated from the others in it.

From the structure analysis, we found that some upland indica accessions, though primarily classified as type indica according to their phenotypes, turn out to be quite complicated in terms of their genomic composition. For example, six accessions (GS190, GS192, GS199, GS200, GS201, GS202) (enclosed in the red rectangular in Figure 2c), previously classified as upland indica according to their phenotypes (e.g., the seed shape, see Additional file 13), turn out to be more close to upland japonica (Figure 2c), indicating that for the upland ecotype, seed shape phenotype could be misleading in distinguishing type indica from type japonica. Besides the six accessions, GS215 and GS233 also have a large genomic proportion close to upland japonica(indicated with blue arrow in Figure 2c), which makes them cluster with japonica in the phylogenetic tree (Figure 1, Additional file 12). And upland indica GS224 (Dular), indicated with a red arrow, has a large proportion of wild rice ancestry, and correspondingly it clusters with the wild rice in the phylogenetic tree (Figure 1, Additional file 12). These results suggest that current upland indica group is a admixture, containing accessions of different origins. They are classified as belong to indica probably because some of their traits are more close to type indica than type japonica. Besides, the 19 upland indica accessions which remain in the indica group in the tree also seem to be polyphyletic, indicating they might originate multiple times and their genetic mechanisms for upland adaptation might be heterogenous. On the contrary, upland japonica accessions that form a monophyletic group are more likely to have similar mechanisms for upland adaption. Considering the well-known hybridization between indica and japonica, it’s probable that upland japonica and some upland indica share similar adaptation genes, while the rest upland indica might be different. Therefore, in the following study on the ecotype differentiated genes, we first use a large sample size by comparing the merged upland and irrigated population to identify genes that show strongest signals and might be shared between upland japonica and some upland indica accessions. Then we mainly focus on the type japonica to find the japonica-specific upland adaptation genes. More data and other experiments would be needed to comprehensively address the adaptation mechanism in the complicated indica group.

### Ecotype differentiated genomic regions (EDRs) in genomes

**Figure 3:**
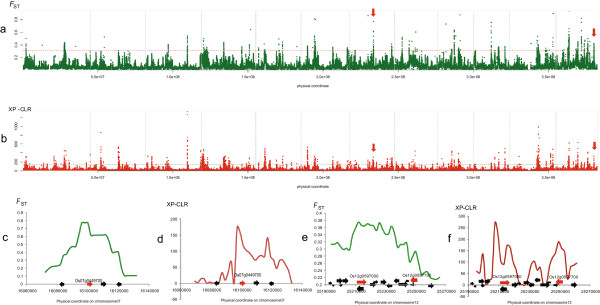
**EDRs scanning. (a)**, *F*_ST_ values were plotted against physical coordinates over the genome; the red horizontal dashed line refers to the top 5‰ threshold. **(b)**, XP-CLR scores were plotted against genomic coordinates; the green horizontal dashed line refers to the top 5‰ threshold. EDRs were obtained by taking the intersection of the EDRs given by the two approaches. The vertical dashed lines in **(a)** and **(b)** indicate the boundaries between two chromosomes; the vertical red arrows indicate the interesting genes shown in detail in **(c - f). (c)** and **(d)** respectively show the *F*_ST_ and XP-CLR values around *Os07g0449700*, an interesting gene annotated as a type A response regulator (indicated using a red arrow). **(e)** and **(f**) respectively show the *F*_ST_ and XP-CLR around *Os12g0597700*, which is similar to WRKY2 and *Os12g0597000* encoding a SOS3-like calcium-binding protein 1 (indicated with red arrows). All of these interesting genes have strong *F*_ST_ and XP-CLR values and are biologically related to upland adaptation in rice.

EDRs probably contain genes that account for the adaptive differentiation. Using a sliding window approach to screen regions with both the top 5‰ *F*_ST_ and cross-population composite likelihood ratio test (XP-CLR) scores (Methods), EDRs were identified with selective signals to narrow down the genes underlying the phenotypic differentiation between upland and irrigated rice (Figure 3). After merging the neighboring EDRs, we eventually identified 74 EDRs in the genome with a median length of 22.7 kb that were differentiated between the upland and irrigated rice (Additional file 14). Within these EDR regions, there are a total of 8980 SNPs (Additional file 15). Compared with the whole genome SNPs used in our analysis, the SNPs in EDRs are significantly enriched in genic regions (Chi-square test, *P* < 2.2e-16) (see Additional file 16). Among the EDR SNPs, 2623 have allele frequency differences larger than 60% between ecotypes, which we subsequently labeled “ecotype differentiated SNPs” (EDSs) (see Additional file 17).

### Ecotype differentiated genes (EDGs) in EDRs

There are 154 genes in the EDRs, which we called EDGs (see Additional file 18). For these EDGs, 74 genes have EDSs in the gene regions and the other 53 genes have EDSs located within 1 kb upstream or downstream regions. By gene enrichment analysis, we found that the EDGs are enriched in several classes (Table 2, Additional file 19), such as lipase containing GDSL domain class (*P* = 7.49e-05), peroxidases (*P* = 1.80e-03), glutathione-related classes (*P* = 5.44e-08), and auxin signaling pathway (*P* = 3.80e-06).

**Table 2:** Interesting EDGs, gene families or pathways

			***P *** **value for enrichment test or gene annotation**	**Potentially impacted phenotypes**
EDGs for the whole population	Enriched gene families or categories	Lipase containing GDSL domain	7.49e-05	Response to drought stress
		Peroxidase	1.80e-03	Response to oxidative stress, drought tolerance
		Glutathione-related genes	5.44e-08	Response to oxidative stress, drought tolerance
		Auxin related genes	3.80e-06	Plant height and roots
	Interesting genes	*Os12g0597000*	SOS3-like calcium binding protein	Response to water deprivation, drought tolerance
		*Os12g0597700*	WRKY DNA binding protein	Drought tolerance
		*Os11g0474600*	terpenoid synthase	Tillering ability
		*Os04g0616400*, *Os04g0616600*	Serine/threonine protein kinase	Drought tolerance
		*Os01g0646000*, *Os06g0184866*, *Os06g0185800*	Pentatricopeptide repeat domain containing protein	Response to oxidative stress, drought tolerance
		*Os07g0449700*	type A response regulator	Plant height and roots
Japonica specific EDGs	Enriched gene families or categories	Mitogen-activated protein kinase	1.37e-14	Response to biotic stress, like blast, and promoting root development
		WRKY genes	0.03	Drought tolerance
		Thioredoxin	0.04	Response to oxidative stress, drought tolerance
	Interesting genes	*Os09g0410500*	homolog to teosinte-branched 1(*tb1*)	Tillering ability

For the EDGs of the whole population and japonica specific EDGs, the enriched gene families and interesting genes in them are shown. And their potentially functional effects are indicated.

The GDSL domain-containing lipase has been reported to be involved in response to abiotic stress, such as drought [25-27], indicating that the genetic differentiation of these genes in upland rice may be relevant to their adaption to dry land. Peroxidases and glutathione-related proteins bear antioxidant activity, another crucial factor in plant adaptation to a drought-like environment [6,7]. Furthermore, auxin, the famous growth-promoting hormone, plays an important role in determining plant architecture, and has been proved to control the formation of adventitious roots [8,28-30]. Accordingly, differences in auxin signaling between upland and irrigated rice could potentially contribute to their different architectures and root systems. Additionally, we also found several individual EDGs of interest that might also be critical in upland adaptation or the phenotypic differentiation between the two ecotypes. *Os07g0449700*, a type-A response regulator, is of special interest (Figure 3c, d). Type-A response regulators belong to ARR gene family induced by cytokinin and play important roles in cytokinin signaling [31]. Quite a few pieces of evidence have shown that ARR genes impact root and shoot development and apparent functional overlap exists among type-A ARRs [31-34]. Moreover, the relevance of type-A ARR gene to the root and shoot phenotypes that are differentiated between upland and irrigated rice has also received experimental support; over-expression of a type-A response regulator in transgenic rice has been shown to cause dwarfness and poorly developed root systems [32]. Hence the ecotype differentiation on this type-A ARR gene, *Os07g0449700*, may be able to explain certain differences of root and plant height phenotypes between upland and irrigated rice. Further reciprocal transgenic experiments between upland and irrigated rice will eventually address the contribution of *Os07g0449700* in the adaptation of upland rice.

Other EDGs were also implied by direct or indirect evidence to be relevant to upland adaptation. *Os12g0597700* (Figure 3e, f) has a WRKY DNA-binding domain and WRKY genes have been well established to be important elements in drought tolerance [35-38]. *Os12g0597000* encodes a SOS3-like calcium-binding protein 1 and its homologs in *Arabidopsis* are related to responses to water deprivation [39]. *Os11g0474600* encodes a terpenoid synthase, and terpenoid plant hormones have been shown to inhibit rice tillering [40]. This gene may, therefore, explain the difference in tiller numbers between upland and irrigated rice. Besides, several pentatricopeptide repeat protein-coding genes (*Os01g0646000*, *Os06g0184866*, *Os06g0185800*) and two serine/threonine protein kinase genes (*Os04g0616400*, *Os04g0616600*) may also be related to drought response as these classes of genes have been reported to be involved in responses to drought/osmotic stress and root development [38,41]. These results illustrate how these 154 EDGs may represent a valuable candidate gene list for investigating the phenotypic differentiation of the two ecotypes and upland rice adaptation.

### EDGs selected in individual populations

**Figure 4:**
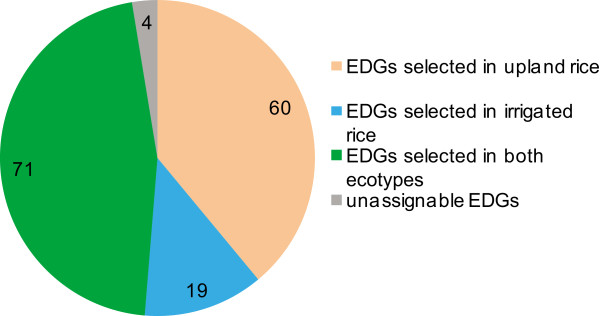
**EDGs population assignment.** Using CLR, 154 EDGs were assigned to specific populations. In total, 60 genes were selected in upland rice, 19 genes in irrigated rice, and 71 genes were selected as probably being in both populations, while four were not able to be assigned to a specific population.

The investigated EDGs indicate selection during the differentiation of the two ecotypes. To further pinpoint in which population the selection occurred, we used Nielsen’s CLR test to identify possible selective sweeps in a single population (see Methods). We identified 661 selective sweep regions in the upland population with a median length of 27 kb, and 351 selective sweep regions in the irrigated population with a median length of 41 kb (Additional file 20). In the selected regions of these upland and irrigated populations, there are 2542 and 1285 genes, respectively. Upland rice then has twice as many genomic regions/genes selected as irrigated rice, indicating that upland rice varieties probably suffered more selection pressure imposed by adaptation or breeding. By overlapping the 154 EDGs with these potentially selected genes, we found that almost all of these selected EDGs (150/154) could be reconfirmed as selected genes using Nielsen’s CLR approach. Among these, 60 were potentially specifically selected in upland rice, 19 were potentially specifically selected in the irrigated population, while 71 were likely selected in both populations (Figure 4, Additional file 18).

### Japonica-specific and Indica-specific EDGs

Genetic architectures of complex traits in rice are often both rich and complicated since they are usually heterogeneous across the subspecies [42]. In light of this, we were also interested in the ecotype-differentiated genes within the type indica or type japonica. As Figure 1 depicts, upland indica have multiple origins, and thus may be genetically heterogeneous in terms of upland adaptation. When investigating the type-specific EDRs, we accordingly mainly focused on the type japonica.

We carried out a similar pipeline to screen the EDRs in type japonica (Additional file 21) and found 52 EDRs with a median length of 24 kb (see Additional file 22). In these EDRs, 169 EDGs were identified (see Additional file 23), among which 47 EDGs are overlapped with the previous EDG list from the total population while the remaining 122 are japonica-specific. For these japonica-specific EDGs, the significantly enriched gene families include mitogen-activated protein kinase (*P* = 1.37e-14), WRKY genes (*P* = 0.03) and thioredoxin coding genes (*P* = 0.04) (Table 2). Previous studies showed that mitogen-activated protein kinase plays an important role in response to biotic stress and root development [11,43]; one japonica-specific EDG of this class is very similar to blast induced kinase, which may then explain why upland rice are generally more blast tolerant than irrigated rice. Moreover, as mentioned previously, WRKY genes and thioredoxin may be critical for drought response. Intriguingly, among our list we found *Os09g0410500*, a homolog to the famous maize-domestication gene, teosinte-branched 1(*tb1*), indicating this gene may also be related to the different tillering abilities displayed by the upland and irrigated types.

For the type indica, because of the admixture of upland indica only 19 upland indica accessions that remain to cluster in the indica group could be used for analysis. But the *F*_ST_ between upland and irrigated accessions in indica is quite lower (0.06) than that in japonica (0.13), which means that a much larger sample would be needed to detect the ecotype differentiation in indica. However, because of the small population size of upland indica accessions in the world it is difficult to collect enough samples so far. Analysis using only the 19 upland indica accessions yield a list of 56 genes identified by intersecting *F*_ST_ and XP-CLR top signals (Additional file 24, Additional file 25). Hence, it is not surprising that none of the 56 genes were overlapped with the previous EDG list from the total population, considering the small sample size and possibly heterogeneous adaptation mechanisms in indica. However, we noticed that nine and four of the genes from the indica XP-CLR top signals overlap with the previous EDGs of the merged populations and EDGs of the japonica population, respectively (Additional file 26, Additional file 27), indicating that some genes related to upland ecotype might be shared among some indica accessions and japonica but are more difficult to detect because of the limited and probable heterogenous samples. More experiments and data are needed to comprehensively address the indica-specific upland adaptation genes.

## Discussion

Largely due to the popularity and fecundity of irrigated rice, there has been limited inquiry about the phenotypes that distinguish upland rice. Some work in this area has been done by Chang et al., who investigated the rate of leaf production, tillering production, plant height, root system and resistance to drought for 11 upland varieties as explanatory factors in the different phenotypes [3]. Likewise, Ono pointed out that Japanese upland varieties are tall and low tillering with broad leaves, long panicles, drought and blast resistant, and have a less efficient utilization of fertilizer [44]. Despite interesting findings, these studies were based on only a few varieties of upland rice. To our knowledge, the sample size of the phenotypic comparisons between upland and irrigated rice in this study represents a very large sample of upland varieties to date and the sample covers most distribution regions of upland rice over the world, providing solid evidence supporting Chang et. al’s observations [3]. Our phenotyping also observed some new features, e.g., the phenomenon that dry land culture increases main root length of upland rice but decreases that of irrigated rice indicates a remarkable superiority of upland rice to adapt to an upland environment.

Beyond our thorough phenotypic analysis, this study, to our knowledge, also provides the first genomic perspective as to the phylogenetics and population structure of current upland rice. Consistent with our classification based on phenotypic characteristics and Glaszmann’s report which indicated most upland cultivars belong to the type japonica [22], phylogenetic analysis revealed that a large proportion of upland accessions belong to the type japonica, which form a monophyly. The population structure inference reveals much less genetic differentiation between the two ecotypes than exists between the type indica-japonica, which could be due to either much more frequent gene flow between ecotypes than that between Indica and Japonica, or recent origin of upland rice.

Though the sequencing depth for each accession used in our study is relatively low, the data set itself is informative enough to address the questions we wanted to address about upland rice adaptation. Firstly, the SNPs identified have a high accuracy (96.4%), even taking into account the low coverage. Secondly, the average sequencing depth for each site in the upland or irrigated population is more than 40×, providing a sufficient sample size for allele frequency estimation. Likewise, only SNP sites with the genotype determined in more than 10 individuals within each population were used for analysis such as calculating allele frequencies, and only SNP sites with no less than 100 individuals’ genotypes were used for estimating population structure. Thirdly, though the genome coverage rate for each accession is not high, for each pair-wise comparison there are enough informative sites for the distance between any two accessions to be precisely computed (see Additional file 28). Thus, as some previous studies have shown, we presented an interesting example substantiating the feasibility of genetic analysis with low coverage population genomic data [45,46].

Except for some QTL mapping efforts, very few reports have touched upon the genetic mechanisms that underlie the adaptive phenotypes of upland rice [47,48]. In this study, we identified some interesting candidate genes or gene families that likely contribute to the upland adaptive traits or phenotypic differentiation between the upland and irrigated ecotypes, not only providing exciting new evidence useful in harnessing the genetic adaptations of upland rice but also demonstrating the efficiency of our methodology. Though pending more experimental evidence to validate the precise phenotypic effects of all the identified alleles, a limited number of genes (around 150) provided a highly feasible list for functional investigation as well as for the upland breeding. Crucially, we would like to stress that our approach most likely caught most of the major genomic regions that account for ecotype differentiation and upland rice adaptation including those specific regions in type japonica, but identifying some indica accession specific adaptation genes still need further work and additional data.

## Conclusions

In summary, we surveyed the phenotypes across a large sample of upland rice in both dry land and irrigated conditions, and by comparing them to irrigated rice in similar conditions we were able to demonstrate some significant phenotypic differentiations between the two ecotypes. Using high-throughput re-sequencing of a large batch of rice accessions, for the first time, to our knowledge, we clarified a single-origin pattern of upland japonica rice but a novel multiple origin pattern in upland indica accessions. With comprehensive population genetic analysis, we identified the ecotype differentiated regions and important selection genes during the differentiation between the upland and irrigated ecotypes, and more poignantly, provided great insights into upland rice adaption and the phenotypic differentiation between the two ecotypes. These findings have important implications in the spread of upland rice varieties in breeding. For example, the genes that likely account for drought resistance can be used to breed highly drought resistant varieties from the existing high-yield irrigated varieties, and concurrently, the genes likely related to tillering ability can be used to enhance the tillering ability of upland rice, a major factor limiting its yield and subsequently its widespread usage.

## Methods

### Investigating phenotypes of upland accessions

To assess the reported phenotypes of upland and irrigated rice, we grew both varieties. Due to the limited amount of seeds for some accessions, only 131 accessions were phenotyped (Additional file 3). Phenotyping was performed both in irrigated and dry land conditions (i.e., preventing submergence in water to simulate the rainfed upland environment). For the irrigated condition, seeds were germinated and seedlings then transplanted to a paddy field with submergence in water for the whole growth and development stage. For the rainfed upland condition, we conducted direct-seeding. To fully simulate the rainfed condition, no irrigation was conducted for the whole growth stage. When the rain came, we released the water to prevent submergence. According to our records, several moderate drought events occurred and caused a few individual plants death in the upland condition. For each accession, we planted 12 individuals in two rows (6 individuals in each row) with the row spacing of 30 centimeters and plant spacing of 20 centimeters. Before the earing period, we counted the tillering numbers. After the plants entered maturity, we surveyed the root weight, main root length, plant height and the yield traits. For each accession, about five individuals were randomly selected and phenotyped (Additional file 4). Because in the rainfed upland condition the percentage of earbearing tiller is close to 100%, in this study tiller number and productive panicle number in the upland condition share the same result.

### The re-sequencing data

The re-sequencing data for the 84 upland and 82 irrigated accessions was reported by our previous work [19]. In our previous study, these data were simply used to test that a selected region containing a key enzyme gene of ABA synthesis was not due to demographic reason. They have neither been used to investigate the population structure for upland rice, nor been used to study the population differentiated regions/genes between the two ecotypes. Here we use these re-sequencing data to further exploit the genetic components that underlie the upland adaptation and phenotypic differentiation between the two ecotypes. The 84 upland and 82 irrigated accessions were obtained from the Yunnan Academy of Agricultural Sciences (YAAS), Yunnan, China. The ecotype information was documented by the genetic resources center of YAAS. Both indica and japonica were selected for either the irrigated or upland groups (Additional file 2). For the irrigated ecotype, we included 42 indica accessions and 40 japonica accessions. For the upland ecotype, we selected 28 indica and 56 japonica accessions since in our genetic resources center most upland rice accessions belong to the type japonica (Additional file 2). The origins of the accessions cover a wide geographic range (Additional file 1) including most major regions of upland rice distribution (South Asia, Southeast Asia, South America, West Africa, etc.). All seeds were sown under irrigated conditions. When the seedlings turned to trefoil-stage, genomic DNA was extracted from the leaves using the CTAB method [49].

### Reads mapping

Our previously obtained re-sequencing data of these upland and irrigated accessions were deposited in the NCBI Short Read Archive under the accession number SRA066116 [19]. The raw reads of the wild rice came from our previous work (NCBI Short Read Archive accession number SRA023116)[21]. The IRGSP 5.0 Nipponbare genome was downloaded from the RAP-DB database (http://rapdblegacy.dna.affrc.go.jp/download/latest/IRGSPb5.fa.masked.gz). We mapped the short reads to the Nipponbare genome using SOAP for each accession separately [20].

### Calling SNPs and SNP annotation

After mapping the reads, we used SOAPsnp -v1.02 to call SNPs for the whole population of the irrigated and upland accessions [50]. A series of filtering was conducted to ensure the quality of the SNPs. For example, the SNPs with a quality value less than 15 or with a nearby copy number > 1.5 were removed. Also we eliminated SNPs sites with depths less than 6 or larger than 300 because sites with too low a depth are prone to sequencing errors while sites with too high depths are likely located within repeat or duplication regions [51]. In order to confirm the accuracy of SNP calling, we randomly selected 60 SNPs, amplified the sequences around them with PCR and subjected them to Sanger sequencing. In our experiment, 56 of the 60 pairs of primers worked. Afterward, genomic annotation was used to assign these SNPs into different categories related to transcription and translation, including genic regions (CDS, introns, and UTRs), promoter regions and intergenic regions using our in-house PERL scripts. SNPs that resulted in premature stop codons, disrupted start/stop codons or splice donor/acceptor sites were defined as radical SNPs.

### Determining SNP genotypes for each accession

For each accession, we extracted the SNP genotype information from the output of SOAPsnp. Again, we conducted a series of quality control procedures to ensure the reliability of the genotype. The consensus files contained information including the best base genotype and its average quality score, the second best base genotype and its average quality score, and the consensus genotype and its quality scores for each site, which can be used as filters [50]. We used very stringent criterion to ensure that only genotypes with a quality score > =20 (with error rate no more than 1%) were taken into consideration. More explicitly, if the average quality score of the best base is > =20 while the average quality score of the second best base is < 20, then that genotype is the best base. If both the average quality score of the best and second best base are > =20, then the genotype is heterozygous. If both the average quality score of the best and second best base are < 20 but the quality score of the consensus genotype is > =20, then the consensus genotype is used. On average 67% of the SNP genotypes were missing for each accession due to the relatively low coverage of our re-sequencing data. However, this missing data will likely not undermine our analyses because the phylogenetic analysis is based on pair-wise distances averaged over many sites and the population genetics analyses are based on allele frequencies. As such, the data are sufficient to estimate the pair-wise distances and allele frequencies. Besides the 166 accessions we re-sequenced, the genotypes of the SNPs for the wild accessions were determined using the same method.

### Constructing the phylogenetic tree

Incorporating the genotype information of wild rice accessions, we use the SNP genotypes of each accession to calculate the pair-wise distances between any two accessions using PHYLIP [52]. We used default parameters to calculate the distances, i.e. using F84 model, a transition/transversion ratio of 2.0 and no Gamma distributed rates across sites. We assumed a uniform mutation rate across the genome and thus gave same weight for different SNPs. Bootstrap with 100 replicates was conducted for the whole genome SNP data set. After the genetic distances were calculated for the 100 replicate data sets, phylogenetic trees were constructed using the Neighbor-Joining (NJ) approach based on the genetic distances [52]. The consensus tree was then built using the built-in ‘consense’ program of PHYLIP with the parameters, ‘strict majority rule’ and ‘trees to be treated as unrooted’.

### Population structure inference

We first conducted a PCA analysis using the procedure as reported [53]. 24890 SNPs with genotype data covering more than 100 accessions was used in the analysis. Moreover, the FRAPPE software [24], which used a frequentist approach to estimate individual admixture, was used to examine the population structure as well. The maximum iteration of EM to run is set as 10000.

### Calculating *F*_ST_

The allele frequencies of SNP alleles were determined based on the accessions’ genotypes. SNPs with less than 10 individuals’ genotype information in one population were discarded when calculating the allele frequencies for that particular population. Based on the allele frequencies, we calculated the ‘*F*_ST_’ using the method described by Nei [54]. In the whole-genome scan, 20-kb sliding windows were used with 2-kb sliding step. By averaging the *F*_ST_ values over SNP sites in each window, *F*_ST_ values for the windows were obtained.

### Scanning selective sweep using composite likelihood approaches

Cross-population composite likelihood ratio test (XP-CLR) and Nielsen’s composite likelihood ratio test (CLR) are two approaches for identifying selective sweep signals using SNP data sets [17,18]. XP-CLR incorporates the allele frequency differentiation patterns between two populations and multilocus frequency spectrum to identify selective sweeps occurring in population differentiation. The Nielsen’s CLR method uses the distribution pattern of allele frequency spectrum in a particular population to search for selective sweep signals in that population. In our analysis, we combined XP-CLR and Nielsen’s CLR using the software XP-CLR and SweepFinder, respectively. In the XP-CLR analysis, we used the allele frequencies of both upland and irrigated populations, calculating XP-CLR scores and picking the top 5‰ regions. When calculating the XP-CLR scores, we used a window size of 0.1 cM, a 2-kb grid size and a maximum SNP number of 150 for each window. By overlapping these regions of top XP-CLR scores with those of top 5‰ *F*_ST_, we obtained a conservative set of ecotype differentiation regions (EDRs) between upland and irrigated rice and the ecotype differentiated genes (EDGs) within these regions. In Nielsen’s CLR analysis, we used allele frequencies of a single population (upland or irrigated population) and found the top 5% grid points with the highest likelihood ratio (LR) values in the genome. Genes within 10 kb of these grid points were considered as the potential selection targets in either rice population [55]. Fine-scale grid points (a grid point at every 1000 nucleotides) were used when calculating the LR values.

### Gene enrichment analysis for the EDGs

Gene enrichment analysis was done using chi-square test. For a specific category of genes, we calculated the gene number in the category within the whole genome using the rice gene annotation information [56]. For example, for the gene category “lipase-containing GDSL domain”, we counted the number of lipase genes annotated with this domain in the rice gene annotation database [56]. The number of genes in this category was also counted in the EDGs. A contingency table was then constructed based on the number of interesting genes in the EDGs, the total gene number of EDGs, the number of interesting genes in the whole genome, and the total gene number in the whole genome. We then conducted the Chi-square test and obtained *P*-values.

### Accession codes

The raw sequence data of the 166 varieties has been deposited into the NCBI Short Read Archive (SRA, http://www.ncbi.nlm.nih.gov/sra/) under accession number SRA066116.

## Abbreviations

XP-CLR: Cross-population composite likelihood ratio; CLR: Composite likelihood ratio; EDR: Ecotype differentiated region; EDG: Genes in EDR; EDS: Ecotype differentiated SNPs.

## Competing interests

The authors declare that they have no competing interests.

## Authors’ contributions

JL, WW, FH designed the project. FH and DT provided the rice materials. HL, JL, LM performed the sequencing, JL, ZG, WH and XL analyzed the data. BL did the phenotyping. JL, SZ, JZ, WH and BL cultivated rice. JL and WW wrote the manuscript. All authors read and approved the final manuscript.

## Supplementary Material

Additional file 1 **Geographic distribution of accessions.** Blue dots show the origins of these accessions, while the dot sizes roughly correspond to the numbers of the accessions from certain areas. Click here for file

Additional file 2 Basic information of the 166 sequenced accessions. Click here for file

Additional file 3 Basic information of the Phenotyped accessions. Click here for file

Additional file 4 The phenotypes of the accessions under rainfed upland condition (a) and irrigated condition (b). Click here for file

Additional file 5 **Phenotype comparison between five upland and five irrigated accessions.** The left five accessions are irrigated rice (in order from left to right, Hongyou 4, Diantun 502, Yunhui 290, Hexi 42, Taizhong 65). The right five accessions are upland rice (in order from left to right IRAT104, CNA4140, Sanlicun, Arias Halus, Dourado). This image indicates that the upland type generally have higher architecture, better developed roots, and fewer tillers. Click here for file

Additional file 6 The correlation between each of the four traits (root weight, maxiaml root length, tiller number and plant height) and the yeild trais (1000-grain weight, number of filled grains per panicle, number of empty grains per panicle and number of productive panicles) under the upland condition. Click here for file

Additional file 7 The maximal main root length (a) and the number of empty grain (b) under irrigated and upland conditions and statistical test. Click here for file

Additional file 8 Mean depth and coverage rate for each accession. Click here for file

Additional file 9 The SNPs for the whole population. Click here for file

Additional file 10 **SNP annotation for the 166 accessions.** a, using genome annotation information we classify the SNPs into intergenic SNPs and SNPs in gene region, which are further classified as SNPs in intron or UTR regions, non-synonymous SNPs and synonymous SNPs. b, SNPs causing radical mutations including disrupting stop/start codons, causing premature stop codons or disupting splicing sites. Click here for file

Additional file 11 Wild rice information. Click here for file

Additional file 12 **Phylogenetics analysis of the upland accessions.** Green, black and orange branches respectively refer to upland, irrigated and wild accessions. The leaf nodes are labeled with the sample number of the accessions. This tree indicates that upland rice seems to have originated multiple times for the type Indica, while upland japonicas probably derived from a single origin. Click here for file

Additional file 13 **Five of the six erroneously classified accessions.** (a), typical indica seed; (b), typical japonica seed; (c) The accessions, GS190(TOS2300), GS192(WAB56-125), GS199(CNA4140), GS200 (GUARANI), GS201(Dourado), GS202(TGR78), were previously classified as upland indica according to their phenotypes (i.e. seed shape, etc.). According to our whole genome phylogenetics analysis, they are more close to upland japonica. GS200 was not photographed due to seeds being unavailable. Click here for file

Additional file 14 **EDR length distribution.** Median length is 22721 bp. Click here for file

Additional file 15 **Annotation of SNPs in EDRs.** In EDRs, there are 8980 SNPs, of which 2409 are located within gene regions. In the gene regions, 241 and 266 SNPs are synonymous and non-synonymous, respectively. Click here for file

Additional file 16 Distribution pattern of SNPs in EDRs. Click here for file

Additional file 17 Ecotype differentiated SNPs (EDS) between upland and irrigated populations. Click here for file

Additional file 18 The 154 EDGs. Click here for file

Additional file 19 Enrichment analysis for interesting gene categories in EDGs and japonica-specific EDGs. Click here for file

Additional file 20 **Length distribution of selective sweep regions in the upland(a) and irrigated(b) population.** Median length is 27002 bp(a) and 41001 bp(b) respectively. Click here for file

Additional file 21 **EDR scanning for type japonica.** (a) *F*_ST_ values between upland japonica and irrigated japonica were plotted against physical coordinates over the genome; the red horizontal dashed line refers to the top 5‰ threshold. (b), XP-CLR scores were plotted against genomic coordinates; the green horizontal dashed line refers to the top 5‰ threshold. EDRs were obtained by taking the intersection of the EDRs given by the two approaches. The vertical dashed lines in (a) and (b) indicate the boundaries between two chromosomes. Click here for file

Additional file 22 **Length distribution of EDRs in type japonica.** Median length is 24019 bp. Click here for file

Additional file 23 Detailed information of the 169 Japonica-specific EDGs. Click here for file

Additional file 24 Detailed information of the 56 Indica-specific EDGs. Click here for file

Additional file 25 **EDR scanning for type indica.** (a) *F*_ST_ values between upland indica and irrigated indica were plotted against physical coordinates over the genome; the red horizontal dashed line refers to the top 5‰ threshold. (b), XP-CLR scores were plotted against genomic coordinates; the green horizontal dashed line refers to the top 5‰ threshold. EDRs were obtained by taking the intersection of the EDRs given by the two approaches. The vertical dashed lines in (a) and (b) indicate the boundaries between two chromosomes. Click here for file

Additional file 26 Nine genes from the indica XP-CLR top signals overlap with the previous EDGs of merged population. Click here for file

Additional file 27 Four genes from the indica XP-CLR top signals overlap with the previous EDGs of the japonica population. Click here for file

Additional file 28 The numbers of informative sites for computing the pair-wise distance between any two accessions. Click here for file
