# Supplementary material for: A genomic perspective on the important genetic mechanisms of upland adaptation of rice
Source: BMC Plant Biol. 2014 Jun 11;14:160. doi: 10.1186/1471-2229-14-160 (PMC4074872; doi:10.1186/1471-2229-14-160)
Supplement: Additional file 1 — Geographic distribution of accessions. Blue dots show the origins of these accessions, while the dot sizes roughly correspond to the numbers of the accessions from certain areas. [file 1471-2229-14-160-S1.docx]

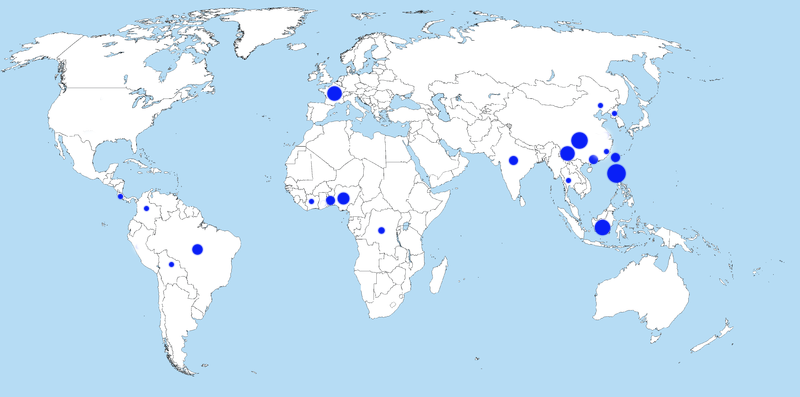


**Additional file 1** **Geographic distribution of accessions.** Blue dots show the origins of these accessions, while the dot sizes roughly correspond to the numbers of the accessions from certain areas.
