# Supplementary material for: A genomic perspective on the important genetic mechanisms of upland adaptation of rice
Source: BMC Plant Biol. 2014 Jun 11;14:160. doi: 10.1186/1471-2229-14-160 (PMC4074872; doi:10.1186/1471-2229-14-160)
Supplement: Additional file 2 — Basic information of the 166 sequenced accessions. [file 1471-2229-14-160-S2.docx]

| Additional file 2: Basic information of the 166 sequenced accessions. | | |  |  |  |
| --- | --- | --- | --- | --- | --- |
| the sample No. | Accession name | YAAS^1^ Serial number | Origin | Type | Ecotype |
| GS001 | IR 22 | FCRI/YAAS 2002 | IRRI^2^ | Indica | irrigated |
| GS002 | IR 29 | FCRI/YAAS 2008 | IRRI | Indica | irrigated |
| GS003 | IR 30 | FCRI/YAAS 2009 | IRRI | Indica | irrigated |
| GS004 | IR 36 | FCRI/YAAS 2011 | IRRI | Indica | irrigated |
| GS005 | IR 43 | FCRI/YAAS 2017 | IRRI | Indica | irrigated |
| GS006 | IR 50 | FCRI/YAAS 2022 | IRRI | Indica | irrigated |
| GS007 | IR 56 | FCRI/YAAS 2024 | IRRI | Indica | irrigated |
| GS008 | IR 64 | FCRI/YAAS 2029 | IRRI | Indica | irrigated |
| GS009 | IR 74 | FCRI/YAAS 2036 | IRRI | Indica | irrigated |
| GS010 | Liuzhiwanmi | FCRI/YAAS 2044 | Guizhou, China | Japonica | irrigated |
| GS013 | Dianyuyihao | FCRI/YAAS 2049 | Dali, Yunnan, China | Japonica | irrigated |
| GS014 | Xian 128 | FCRI/YAAS 2051 | Fujian, China | Indica | irrigated |
| GS015 | Shuanggui 36 | FCRI/YAAS 2054 | Guangdong, China | Indica | irrigated |
| GS016 | Xinguiai | FCRI/YAAS 2058 | Guangdong, China | Indica | irrigated |
| GS017 | Jinmazhan | FCRI/YAAS 2072 | Guizhou, China | Indica | irrigated |
| GS019 | Xiushui 11 | FCRI/YAAS 2080 | Jiangsu, China | Japonica | irrigated |
| GS020 | Jingjing 1 | FCRI/YAAS 2094 | Yunnan | Japonica | irrigated |
| GS025 | Shennong 1033 | FCRI/YAAS 2120 | Shenyang | Japonica | irrigated |
| GS027 | Liao 942 | FCRI/YAAS 2170 |  | Japonica | irrigated |
| GS028 | Shennong 8712 | FCRI/YAAS 2175 |  | Japonica | irrigated |
| GS029 | Miyang 46 | FCRI/YAAS 2189 |  | Indica | irrigated |
| GS030 | Minghui 63 | FCRI/YAAS 2190 |  | Indica | irrigated |
| GS033 | Danjing 5 | FCRI/YAAS 2196 |  | Japonica | irrigated |
| GS034 | Zhuzhan | FCRI/YAAS 2201 |  | Indica | irrigated |
| GS036 | Dianchao 5 | FCRI/YAAS 2254 |  | Indica | irrigated |
| GS037 | Dianchao 7 | FCRI/YAAS 2283 | Yunnan, China | Indica | irrigated |
| GS038 | Diantun 502 | FCRI/YAAS 2284 | Ruili, Yunnan, China | Indica | irrigated |
| GS050 | Dianxi 1 | FCRI/YAAS 2384 | Zhoujiawu^3^, China | Japonica | irrigated |
| GS053 | Taizhong 65 | FCRI/YAAS 2387 | Zhoujiawu, China | Japonica | irrigated |
| GS054 | Taizhong 150 | FCRI/YAAS 2388 | Zhoujiawu, China | Japonica | irrigated |
| GS055 | Taizhong 179 | FCRI/YAAS 2389 | Zhoujiawu, China | Japonica | irrigated |
| GS056 | Tainan 1 | FCRI/YAAS 2390 | Zhoujiawu, China | Japonica | irrigated |
| GS057 | Gaoxiong 22 | FCRI/YAAS 2391 | Zhoujiawu, China | Japonica | irrigated |
| GS059 | Dianjingyou 1 | FCRI/YAAS 2394 |  | Japonica | irrigated |
| GS060 | Dianyu 2 | FCRI/YAAS 2412 | IRRI | Japonica | irrigated |
| GS061 | RD 23 | FCRI/YAAS 2414 | IRRI | Indica | irrigated |
| GS062 | Yundao 1 | FCRI/YAAS 2415 | IRRI | Japonica | irrigated |
| GS064 | Hexi 35 | FCRI/YAAS 2419 | IRRI | Japonica | irrigated |
| GS065 | Hexi 39 | FCRI/YAAS 2421 | IRRI | Japonica | irrigated |
| GS068 | Fengdao 14 | FCRI/YAAS 2551 | IRRI | Japonica | irrigated |
| GS069 | Dianxi 4 | FCRI/YAAS 3070 | YAAS | Japonica | irrigated |
| GS070 | Chujing 3 | FCRI/YAAS 3071 | YAAS | Japonica | irrigated |
| GS071 | Chujing 24 | FCRI/YAAS 3073 | YAAS | Japonica | irrigated |
| GS072 | Chujing 25 | FCRI/YAAS 3074 | YAAS | Japonica | irrigated |
| GS074 | Diantun 502 | FCRI/YAAS 3076 | YAAS | Indica | irrigated |
| GS076 | Hongyou 3 | FCRI/YAAS 3078 | YAAS | Indica | irrigated |
| GS077 | Hongyou 4 | FCRI/YAAS 3079 | YAAS | Indica | irrigated |
| GS079 | Dianjingyou 4 | FCRI/YAAS 3090 | YAAS | Japonica | irrigated |
| GS080 | Dianchao 6 | FCRI/YAAS 3095 | YAAS | Japonica | irrigated |
| GS081 | Dianchao 8 | FCRI/YAAS 3097 | YAAS | Japonica | irrigated |
| GS082 | Yunhui 188 | FCRI/YAAS 3099 | YAAS | Indica | irrigated |
| GS084 | Nipponbare | FCRI/YAAS 3108 | YAAS | Japonica | irrigated |
| GS085 | 2-32B | FCRI/YAAS 3109 | YAAS | Indica | irrigated |
| GS086 | Zhonghua 11 | FCRI/YAAS 3111 | YAAS | Japonica | irrigated |
| GS087 | Kitaaki | FCRI/YAAS 3112 | YAAS | Japonica | irrigated |
| GS088 | Xinzhu 62 | FCRI/YAAS 3113 | YAAS | Japonica | irrigated |
| GS089 | Gaoxiong 53 | FCRI/YAAS 3118 | YAAS | Japonica | irrigated |
| GS091 | IR 72 | FCRI/YAAS 3124 | YAAS | Indica | irrigated |
| GS093 | Dianrui 462 | FCRI/YAAS 3128 | YAAS | Indica | irrigated |
| GS096 | Hongzaosheng |  |  | Japonica | irrigated |
| GS097 | Banna21 |  |  | Indica | irrigated |
| GS098 | Toride 1 |  |  | Japonica | irrigated |
| GS099 | Dee-goo-woo-gen |  |  | Indica | irrigated |
| GS100 | Wendao 5 |  |  | Indica | irrigated |
| GS101 | Denong 205 |  |  | Indica | irrigated |
| GS102 | Shenglixian |  |  | Indica | irrigated |
| GS103 | Menggudao |  |  | Japonica | irrigated |
| GS104 | Zhuzhan |  |  | Indica | irrigated |
| GS105 | 8-126 |  |  | Japonica | irrigated |
| GS106 | Nantehao |  |  | Indica | irrigated |
| GS107 | Aizizhan |  |  | Indica | irrigated |
| GS108 | Peta |  |  | Indica | irrigated |
| GS109 | Lijiangxintuanheigu |  |  | Japonica | irrigated |
| GS111 | Dianrui 449 |  |  | Indica | irrigated |
| GS112 | IR 20 |  |  | Indica | irrigated |
| GS113 | IR 24 |  |  | Indica | irrigated |
| GS114 | IR 28 |  |  | Indica | irrigated |
| GS116 | IR 38 |  |  | Indica | irrigated |
| GS117 | IR 40 |  |  | Indica | irrigated |
| GS119 | Yunjing 9 |  |  | Japonica | irrigated |
| GS122 | Yunjing 219c |  |  | Japonica | irrigated |
| GS123 | Yunjing 78-215c |  |  | Japonica | irrigated |
| GS126 | Xiaobaigu | FCRI/YAAS 1009 | Lancang, Yunnan, China | Japonica | upland |
| GS127 | Aoyong | FCRI/YAAS 1012 | Lancang, Yunnan, China | Japonica | upland |
| GS128 | Zhaxima | FCRI/YAAS 1024 | Lancang, Yunnan, China | Japonica | upland |
| GS129 | Xihonggu | FCRI/YAAS 1046 | Lancang, Yunnan, China | Japonica | upland |
| GS130 | Haohaohao | FCRI/YAAS 1051 | Lancang, Yunnan, China | Japonica | upland |
| GS131 | Banli 1 | FCRI/YAAS 1058 | Lancang, Yunnan, China | Japonica | upland |
| GS132 | Pangxiegu | FCRI/YAAS 1067 | Lancang, Yunnan, China | Japonica | upland |
| GS133 | Bayuenuo | FCRI/YAAS 1068 | Lancang, Yunnan, China | Japonica | upland |
| GS134 | Lancangdabaigu | FCRI/YAAS 1069 | Lancang, Yunnan, China | Japonica | upland |
| GS135 | Landigu | FCRI/YAAS 1073 | Lancang, Yunnan, China | Japonica | upland |
| GS136 | Huangpigu | FCRI/YAAS 1113 | Lancang, Yunnan, China | Japonica | upland |
| GS137 | Bayuenuo | FCRI/YAAS 1135 | Lancang, Yunnan, China | Japonica | upland |
| GS138 | Mengwanggu | FCRI/YAAS 1188 | Lancang, Yunnan, China | Japonica | upland |
| GS140 | Mazigu | FCRI/YAAS 1196 | Lancang, Yunnan, China | Japonica | upland |
| GS141 | Hongzaogu | FCRI/YAAS 1217 | Lancang, Yunnan, China | Japonica | upland |
| GS142 | Haodali | FCRI/YAAS 1228 | Menghai, Yunnan, China | Japonica | upland |
| GS145 | Zigu | FCRI/YAAS 1255 | Shizong, Yunnan, China | Japonica | upland |
| GS146 | Dahonggu | FCRI/YAAS 1260 | Shizong, Yunnan, China | Japonica | upland |
| GS148 | Jiaojiajingdabaigu | FCRI/YAAS 1272 | Shizong, Yunnan, China | Japonica | upland |
| GS149 | Heijugu | FCRI/YAAS 1279 | Shizong, Yunnan, China | Japonica | upland |
| GS150 | Dahongbaigu | FCRI/YAAS 1285 | Gengma, Yunnan, China | Japonica | upland |
| GS151 | Beizichangmao | FCRI/YAAS 1289 | Gengma, Yunnan, China | Japonica | upland |
| GS152 | Xiaobaigu | FCRI/YAAS 1292 | Gengma, Yunnan, China | Japonica | upland |
| GS154 | Yangbiguangkeludao | FCRI/YAAS 1316 | Yangbi, Yunnan, China | Japonica | upland |
| GS155 | Sanlicun | FCRI/YAAS 1323 | Guangnan, Yunnan, China | Japonica | upland |
| GS156 | Boyegu | FCRI/YAAS 1329 | Lvchun, Yunnan, China | Japonica | upland |
| GS157 | Liandaogu | FCRI/YAAS 1334 | Lvchun, Yunnan, China | Japonica | upland |
| GS158 | Digunuo | FCRI/YAAS 1338 | Honghe, Yunnan, China | Japonica | upland |
| GS159 | Lunanhangu | FCRI/YAAS 1343 | Lunan, Yunnan, China | Japonica | upland |
| GS160 | Dabaigu | FCRI/YAAS 1354 | Yuanyang, China | Japonica | upland |
| GS161 | Sanbang 70 Luo | FCRI/YAAS 1356 | Tengchong, Yunnan, China | Japonica | upland |
| GS162 | Zhaluoge | FCRI/YAAS 1370 | Menglian, Yunnan, China | Japonica | upland |
| GS163 | Caopigu | FCRI/YAAS 1389 | Mojiang, Yunnan, China | Japonica | upland |
| GS164 | Shanekuai | FCRI/YAAS 1390 | Mojiang, Yunnan, China | Japonica | upland |
| GS165 | Huangkehongmangjingzhan | FCRI/YAAS 1401 | Guizhou, China | Japonica | upland |
| GS167 | Qinglonghanniangu | FCRI/YAAS 1412 | Guizhou, China | Indica | upland |
| GS168 | Wangmonianhangu | FCRI/YAAS 1418 | Guizhou, China | Indica | upland |
| GS169 | Wangmozihuake | FCRI/YAAS 1427 | Guizhou, China | Indica | upland |
| GS171 | Baihandao | FCRI/YAAS 1453 | Guizhou, China | Japonica | upland |
| GS172 | Anshunbaimihannuo | FCRI/YAAS 1467 | Guizhou, China | Japonica | upland |
| GS173 | Qinglonghuangnuo | FCRI/YAAS 1492 | Guizhou, China | Japonica | upland |
| GS175 | cehengguangkexianjing | FCRI/YAAS 1509 | Guizhou, China | Japonica | upland |
| GS176 | Dejianggoudiantou | FCRI/YAAS 1538 | Guizhou, China | Japonica | upland |
| GS177 | Congjiangxunwenjiu | FCRI/YAAS 1547 | Guizhou, China | Japonica | upland |
| GS178 | Taiwanludao 1 | FCRI/YAAS 1549 | Taiwan | Japonica | upland |
| GS179 | Cehengguangkenuo | FCRI/YAAS 1568 | Guizhou, China | Japonica | upland |
| GS180 | Xingrenhonghanniangu | FCRI/YAAS 1587 | Guizhou, China | Japonica | upland |
| GS181 | Digannuo | FCRI/YAAS 1633 | Guangdong, China | Japonica | upland |
| GS182 | IAC 25 | FCRI/YAAS 5509 | IITA^4^ (Nigeria) | Japonica | upland |
| GS186 | ITA 117 | FCRI/YAAS 5515 | IITA (Nigeria) | Japonica | upland |
| GS187 | ITA 118 | FCRI/YAAS 5516 | IITA (Nigeria) | Japonica | upland |
| GS190 | TOS 2300 | FCRI/YAAS 5553 | IITA (Nigeria) | Indica | upland |
| GS192 | WAB56-125 | FCRI/YAAS 5793 | WARDA^5^ (western Africa) | Indica | upland |
| GS193 | WAB56-50 | FCRI/YAAS 5794 | WARDA (western Africa) | Japonica | upland |
| GS199 | CNA 4140 | FCRI/YAAS 5815 | Brazil | Indica | upland |
| GS200 | GUARANI | FCRI/YAAS 5831 | Brazil | Indica | upland |
| GS201 | Dourado | FCRI/YAAS 5851 | Bolivia | Indica | upland |
| GS202 | TGR 78 | FCRI/YAAS 5852 | Duoge, Yunnan, China | Indica | upland |
| GS204 | CIRAD 391 | FCRI/YAAS 5854 | France | Japonica | upland |
| GS208 | IRAT 12 | FCRI/YAAS 5877 | France | Indica | upland |
| GS209 | IRAT 104 | FCRI/YAAS 5885 | France | Japonica | upland |
| GS210 | IRAT 109 | FCRI/YAAS 5891 | France | Japonica | upland |
| GS211 | IRAT 110 | FCRI/YAAS 5895 | France | Japonica | upland |
| GS212 | IRAT 112 | FCRI/YAAS 5898 | France | Japonica | upland |
| GS214 | Azucena | FCRI/YAAS 6044 | Philippines | Japonica | upland |
| GS215 | CICA 6 | FCRI/YAAS 6048 | Philippines | Indica | upland |
| GS216 | UPL RI-5 | FCRI/YAAS 6052 | Philippines | Indica | upland |
| GS217 | CICA 9 | FCRI/YAAS 6060 | Columbia | Indica | upland |
| GS218 | CR 5272 | FCRI/YAAS 6068 | Costa Rica | Indica | upland |
| GS219 | KMP 34 | FCRI/YAAS 6077 | Côte d'Ivoire | Indica | upland |
| GS221 | Nam Reo | FCRI/YAAS 6124 | Thailand | Japonica | upland |
| GS224 | Dular | FCRI/YAAS 6147 | India | Indica | upland |
| GS226 | Salumpikit | FCRI/YAAS 6180 | India | Indica | upland |
| GS228 | B2997C-7B-4-2-1 | FCRI/YAAS 6186 | Indonesia | Indica | upland |
| GS229 | B3619C-7B-8-1-4 | FCRI/YAAS 6187 | Indonesia | Indica | upland |
| GS230 | B4801F-MR5 | FCRI/YAAS 6190 | Indonesia | Indica | upland |
| GS231 | B5524F-ST-30-14 | FCRI/YAAS 6191 | Indonesia | Indica | upland |
| GS232 | B6128-64-0-TB-TB-0-6-0-0 | FCRI/YAAS 6193 | Indonesia | Indica | upland |
| GS233 | B6136-3-TB-0-1-5 | FCRI/YAAS 6195 | Indonesia | Indica | upland |
| GS234 | B6144 | FCRI/YAAS 6198 | Indonesia | Indica | upland |
| GS235 | B6144F-MR-6 | FCRI/YAAS 6199 | Indonesia | Indica | upland |
| GS236 | B6824E-TB-3 | FCRI/YAAS 6203 | Indonesia | Indica | upland |
| GS243 | Jiangchengkugu |  |  | Indica | upland |
| GS244 | Luyin 46 |  |  | Indica | upland |
| ^1^ refers to the Yunnan Academy of Agriculture Sciences  ^2^ refers to the International Rice Research Institute  ^3^ refers to a Chinese breeder in Yunnan Academy of Agricultural Sciences  ^4^ refers to the Agricultural Research for Development in Africa  ^5^ refers to the West Africa Rice Development Association | | | | | |
