# Supplementary material for: A genomic perspective on the important genetic mechanisms of upland adaptation of rice
Source: BMC Plant Biol. 2014 Jun 11;14:160. doi: 10.1186/1471-2229-14-160 (PMC4074872; doi:10.1186/1471-2229-14-160)
Supplement: Additional file 3 — Basic information of the Phenotyped accessions. [file 1471-2229-14-160-S3.docx]

| Additional file 3: Phenotyped accessions. | | |
| --- | --- | --- |
| Accession name | the sample No. | Ecotype |
| Dee-goo-woo-gen | GS099 | irrigated |
| Guichao 2 | GS110 | irrigated |
| IR 64 | GS008 | irrigated |
| Chujing 25 | GS072 | irrigated |
| Taizhong 150 | GS054 | irrigated |
| Denong 202 | GS073 | irrigated |
| Hongyou 2 | GS075 | irrigated |
| Chugeng 27 |  | irrigated |
| Hongyou 3 | GS076 | irrigated |
| IR 22 | GS001 | irrigated |
| IR 29 | GS002 | irrigated |
| IR 36 | GS004 | irrigated |
| IR 50 | GS006 | irrigated |
| IR 56 | GS007 | irrigated |
| IR 74 | GS009 | irrigated |
| Liuzhiwanmi | GS010 | irrigated |
| Xian 128 | GS014 | irrigated |
| Shuanggui 36 | GS015 | irrigated |
| Xinguiai | GS016 | irrigated |
| Jinmazhan | GS017 | irrigated |
| Liao 942 | GS027 | irrigated |
| Miyang 46 | GS029 | irrigated |
| Danjing 5 | GS033 | irrigated |
| 8-126 | GS105 | irrigated |
| Menggudao | GS103 | irrigated |
| Denong 205 | GS101 | irrigated |
| Xueheaizao |  | irrigated |
| Xinzhu 56 |  | irrigated |
| Tainan 1 | GS056 | irrigated |
| Gaoxiong 22 | GS057 | irrigated |
| Xinzhu 62 | GS088 | irrigated |
| Dianjingyou 1 | GS059 | irrigated |
| Dianyu 2 | GS060 | irrigated |
| 2-32B | GS085 | irrigated |
| RD 23 | GS061 | irrigated |
| Hexi 22 |  | irrigated |
| Hexi 39 | GS065 | irrigated |
| Hexi 42 | GS066 | irrigated |
| Fengdao 14 | GS068 | irrigated |
| Chujing 3 | GS070 | irrigated |
| Chujing 24 | GS071 | irrigated |
| Diantun 502 | GS074 | irrigated |
| Hongyou 4 | GS077 | irrigated |
| Yunhui 290 | GS078 | irrigated |
| Dianjingyou 4 | GS079 | irrigated |
| Dianchao 8 | GS081 | irrigated |
| Hongzaosheng | GS096 | irrigated |
| Banna 21 | GS097 | irrigated |
| Nantehao | GS106 | irrigated |
| Aizizhan | GS107 | irrigated |
| Wendao 5 | GS100 | irrigated |
| Denong 205 | GS101 | irrigated |
| Shenglixian | GS102 | irrigated |
| Menggudao | GS103 | irrigated |
| Dianrui 449 | GS111 | irrigated |
| IR 20 | GS112 | irrigated |
| Jiangchengkugu | GS243 | upland |
| Qinglonghanniangu | GS167 | upland |
| TGR 78 | GS202 | upland |
| IRAT 12 | GS208 | upland |
| CICA 6 | GS215 | upland |
| CICA 9 | GS217 | upland |
| Luyin 46 | GS244 | upland |
| Huangkehongmangjingzhan | GS165 | upland |
| AZUCENA | GS214 | upland |
| Jaya | GS225 | upland |
| Arias Halus | GS227 | upland |
| Dourado | GS201 | upland |
| CNA 4140 | GS199 | upland |
| IRAT 104 | GS209 | upland |
| Sanlicun | GS155 | upland |
| CAIAPO | GS196 | upland |
| CNA 4120 | GS197 | upland |
| CIRAD 392 | GS205 | upland |
| Xingrenhonghanniangu | GS180 | upland |
| Xiaobaigu | GS152 | upland |
| Aoyong | GS127 | upland |
| Zhaxima | GS128 | upland |
| Haohaohao | GS130 | upland |
| Bayuenuo | GS137 | upland |
| Lancangdabaigu | GS134 | upland |
| Landigu | GS135 | upland |
| Huangpigu | GS136 | upland |
| Bayuenuo | GS137 | upland |
| Mengwanggu | GS138 | upland |
| Mazigu | GS140 | upland |
| Hongzaogu | GS141 | upland |
| Haodali | GS142 | upland |
| Zigu | GS145 | upland |
| Dahonggu | GS146 | upland |
| Jiaojiajingdabaigu | GS148 | upland |
| Heijugu | GS149 | upland |
| Dahongbaigu | GS150 | upland |
| Xiaobaigu | GS152 | upland |
| Yangbiguangkeludao | GS154 | upland |
| Boyegu | GS156 | upland |
| Liandaogu | GS157 | upland |
| Lunanhangu | GS159 | upland |
| Dabaigu | GS160 | upland |
| Sanbang 70 Luo | GS161 | upland |
| Zhaluoge | GS162 | upland |
| Caopigu | GS163 | upland |
| Shanekuai | GS164 | upland |
| Wangmonianhangu | GS168 | upland |
| Baihandao | GS171 | upland |
| cehengguangkexianjing | GS175 | upland |
| Dejianggoudiantou | GS176 | upland |
| Taiwanludao 1 | GS178 | upland |
| Cehengguangkenuo | GS179 | upland |
| Digannuo | GS181 | upland |
| IAC 25 | GS182 | upland |
| ITA 118 | GS187 | upland |
| TOS 2300 | GS190 | upland |
| WAB56-125 | GS192 | upland |
| WAB56-50 | GS193 | upland |
| GUARANI | GS200 | upland |
| CIRAD 391 | GS204 | upland |
| IRAT 110 | GS211 | upland |
| IRAT 112 | GS212 | upland |
| Azucena | GS214 | upland |
| UPL RI-5 | GS216 | upland |
| CR 5272 | GS218 | upland |
| KMP 34 | GS219 | upland |
| B3619C-7B-8-1-4 | GS229 | upland |
| B4801F-MR5 | GS230 | upland |
| B5524F-ST-30-14 | GS231 | upland |
| B6128-64-0-TB-TB-0-6-0-0 | GS232 | upland |
| B6136-3-TB-0-1-5 | GS233 | upland |
| B6144 | GS234 | upland |
| B6144F-MR-6 | GS235 | upland |
| B6824E-TB-3 | GS236 | upland |
