# Supplementary material for: A genomic perspective on the important genetic mechanisms of upland adaptation of rice
Source: BMC Plant Biol. 2014 Jun 11;14:160. doi: 10.1186/1471-2229-14-160 (PMC4074872; doi:10.1186/1471-2229-14-160)
Supplement: Additional file 5 — Phenotype comparison between five upland and five irrigated accessions. The left five accessions are irrigated rice (in order from left to right, Hongyou 4, Diantun 502, Yunhui 290, Hexi 42, Taizhong 65). The right five accessions are upland rice (in order from left to right IRAT104, CNA4140, Sanlicun, Arias Halus, Dourado). This image indicates that the upland type generally have higher architecture, better developed roots, and fewer tillers. [file 1471-2229-14-160-S5.docx]

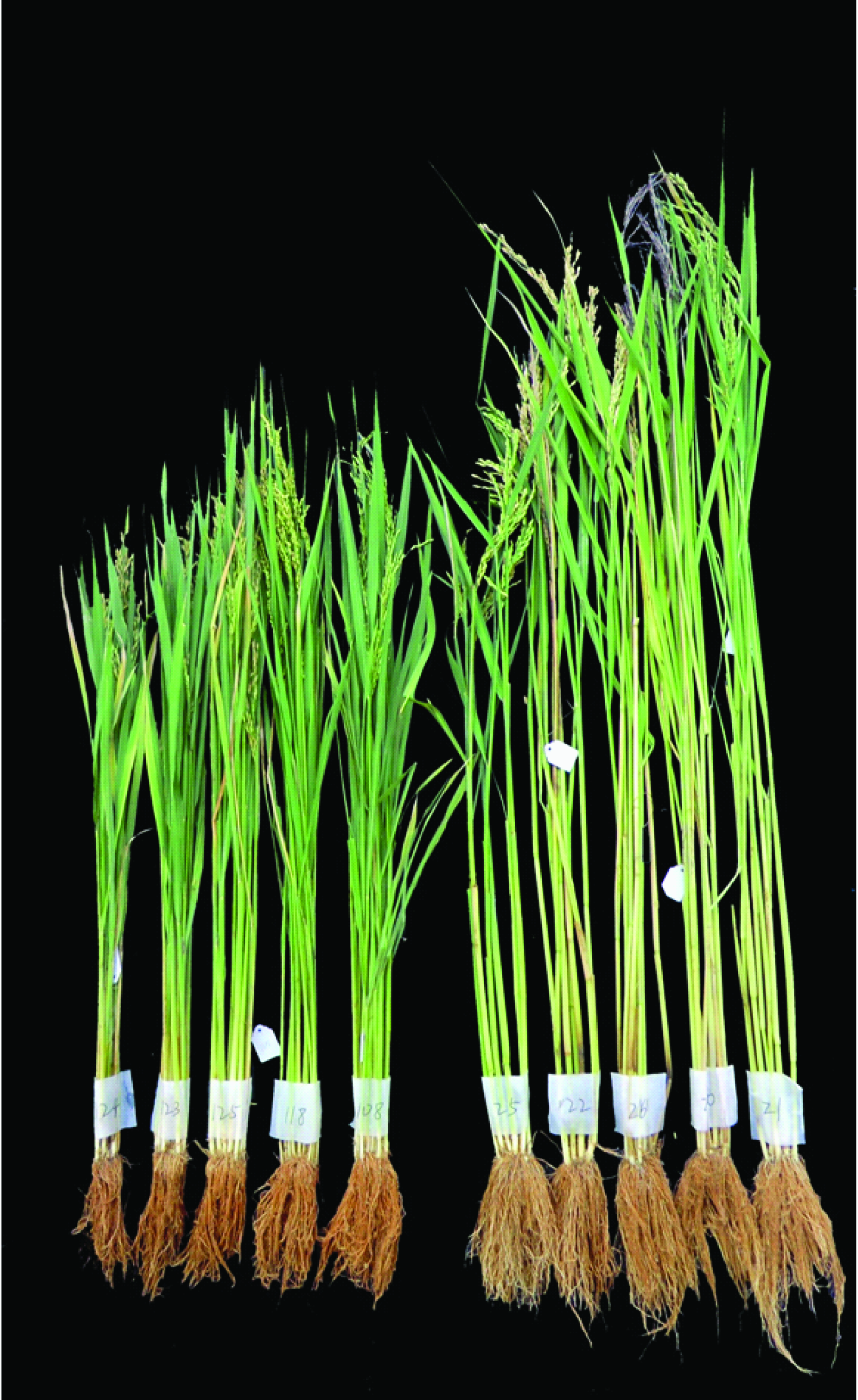


**Additional file 5 Phenotype comparison between five upland and five irrigated accessions.** The left five accessions are irrigated rice (in order from left to right, Hongyou 4, Diantun 502, Yunhui 290, Hexi 42, Taizhong 65). The right five accessions are upland rice (in order from left to right IRAT104, CNA4140, Sanlicun, Arias Halus, Dourado). This image indicates that the upland type generally have higher architecture, better developed roots, and fewer tillers.
