# Supplementary material for: A genomic perspective on the important genetic mechanisms of upland adaptation of rice
Source: BMC Plant Biol. 2014 Jun 11;14:160. doi: 10.1186/1471-2229-14-160 (PMC4074872; doi:10.1186/1471-2229-14-160)
Supplement: Additional file 8 — Mean depth and coverage rate for each accession. [file 1471-2229-14-160-S8.docx]

| Additional file 8: Mean depth and coverage rate for each accession. | | |
| --- | --- | --- |
| the sample No. | mean depth | coverage |
| GS001 | 0.40 | 25.56% |
| GS002 | 0.44 | 26.23% |
| GS003 | 0.43 | 26.54% |
| GS004 | 0.41 | 25.02% |
| GS005 | 0.40 | 24.11% |
| GS006 | 0.39 | 23.90% |
| GS007 | 0.41 | 25.75% |
| GS008 | 0.48 | 28.20% |
| GS009 | 0.43 | 25.71% |
| GS010 | 0.66 | 37.68% |
| GS013 | 0.67 | 39.14% |
| GS014 | 0.45 | 26.57% |
| GS015 | 0.53 | 28.89% |
| GS016 | 0.43 | 24.60% |
| GS017 | 0.37 | 23.10% |
| GS019 | 0.75 | 41.46% |
| GS020 | 0.55 | 38.99% |
| GS025 | 0.66 | 37.16% |
| GS027 | 0.70 | 41.20% |
| GS028 | 0.72 | 39.72% |
| GS029 | 0.38 | 23.60% |
| GS030 | 0.44 | 25.08% |
| GS033 | 0.70 | 39.01% |
| GS034 | 0.35 | 21.51% |
| GS036 | 0.28 | 23.81% |
| GS037 | 0.38 | 23.11% |
| GS038 | 0.45 | 26.37% |
| GS050 | 0.69 | 37.87% |
| GS053 | 0.68 | 38.85% |
| GS054 | 0.62 | 36.37% |
| GS055 | 0.63 | 36.04% |
| GS056 | 0.73 | 40.26% |
| GS057 | 0.58 | 34.43% |
| GS059 | 0.73 | 39.67% |
| GS060 | 0.62 | 34.24% |
| GS061 | 0.33 | 20.66% |
| GS062 | 0.74 | 40.79% |
| GS064 | 0.69 | 38.36% |
| GS065 | 0.64 | 37.10% |
| GS068 | 0.69 | 39.20% |
| GS069 | 0.58 | 34.08% |
| GS070 | 0.47 | 35.70% |
| GS071 | 0.64 | 37.57% |
| GS072 | 0.68 | 37.39% |
| GS074 | 0.42 | 26.63% |
| GS076 | 0.41 | 24.62% |
| GS077 | 0.42 | 27.54% |
| GS079 | 0.66 | 37.97% |
| GS080 | 0.62 | 36.17% |
| GS081 | 0.63 | 37.58% |
| GS082 | 0.51 | 31.63% |
| GS084 | 0.59 | 34.94% |
| GS085 | 0.50 | 26.56% |
| GS086 | 0.50 | 31.36% |
| GS087 | 0.62 | 34.90% |
| GS088 | 0.65 | 37.48% |
| GS089 | 0.60 | 34.81% |
| GS091 | 0.38 | 24.80% |
| GS093 | 0.43 | 25.52% |
| GS096 | 0.67 | 39.25% |
| GS097 | 0.42 | 26.56% |
| GS098 | 0.59 | 36.07% |
| GS099 | 0.38 | 24.20% |
| GS100 | 0.39 | 25.75% |
| GS101 | 0.42 | 25.91% |
| GS102 | 0.36 | 23.65% |
| GS103 | 0.66 | 38.34% |
| GS104 | 0.27 | 21.48% |
| GS105 | 0.67 | 38.25% |
| GS106 | 0.44 | 26.60% |
| GS107 | 0.47 | 27.93% |
| GS108 | 0.37 | 22.54% |
| GS109 | 0.64 | 36.62% |
| GS111 | 0.38 | 22.95% |
| GS112 | 0.38 | 24.29% |
| GS113 | 0.37 | 23.10% |
| GS114 | 0.36 | 25.51% |
| GS116 | 0.45 | 26.28% |
| GS117 | 0.40 | 25.28% |
| GS119 | 0.61 | 36.64% |
| GS122 | 0.63 | 36.54% |
| GS123 | 0.52 | 34.23% |
| GS126 | 0.31 | 20.93% |
| GS127 | 0.34 | 23.01% |
| GS128 | 0.36 | 23.54% |
| GS129 | 0.36 | 24.00% |
| GS130 | 0.30 | 20.51% |
| GS131 | 0.33 | 22.84% |
| GS132 | 0.34 | 22.36% |
| GS133 | 0.34 | 23.61% |
| GS134 | 0.33 | 22.68% |
| GS135 | 0.33 | 21.76% |
| GS136 | 0.34 | 23.03% |
| GS137 | 0.35 | 23.24% |
| GS138 | 0.32 | 22.01% |
| GS140 | 0.32 | 21.78% |
| GS141 | 0.32 | 21.96% |
| GS142 | 0.30 | 21.24% |
| GS145 | 0.30 | 20.64% |
| GS146 | 0.31 | 21.58% |
| GS148 | 0.33 | 22.67% |
| GS149 | 0.27 | 19.52% |
| GS150 | 0.31 | 21.54% |
| GS151 | 0.29 | 20.78% |
| GS152 | 0.48 | 31.00% |
| GS154 | 0.31 | 20.39% |
| GS155 | 0.27 | 18.65% |
| GS156 | 0.44 | 27.94% |
| GS157 | 0.36 | 23.50% |
| GS158 | 0.35 | 23.67% |
| GS159 | 0.37 | 23.83% |
| GS160 | 0.33 | 21.96% |
| GS161 | 0.36 | 23.59% |
| GS162 | 0.35 | 23.40% |
| GS163 | 0.41 | 25.84% |
| GS164 | 0.37 | 24.74% |
| GS165 | 0.36 | 23.91% |
| GS167 | 0.73 | 36.34% |
| GS168 | 0.77 | 35.99% |
| GS169 | 0.77 | 37.33% |
| GS171 | 0.34 | 23.38% |
| GS172 | 0.35 | 23.70% |
| GS173 | 0.36 | 28.34% |
| GS175 | 0.38 | 24.82% |
| GS176 | 0.34 | 22.38% |
| GS177 | 0.38 | 24.41% |
| GS178 | 0.35 | 23.49% |
| GS179 | 0.31 | 21.22% |
| GS180 | 0.32 | 22.02% |
| GS181 | 0.31 | 21.40% |
| GS182 | 0.36 | 24.95% |
| GS186 | 0.36 | 24.09% |
| GS187 | 0.33 | 22.42% |
| GS190 | 0.93 | 46.45% |
| GS192 | 0.83 | 42.93% |
| GS193 | 0.34 | 23.57% |
| GS199 | 0.94 | 45.94% |
| GS200 | 0.79 | 41.62% |
| GS201 | 0.96 | 46.58% |
| GS202 | 0.89 | 44.30% |
| GS204 | 0.37 | 24.79% |
| GS208 | 0.76 | 35.95% |
| GS209 | 0.33 | 22.08% |
| GS210 | 0.31 | 21.57% |
| GS211 | 0.34 | 22.79% |
| GS212 | 0.35 | 23.47% |
| GS214 | 0.31 | 22.31% |
| GS215 | 0.87 | 42.44% |
| GS216 | 0.75 | 37.33% |
| GS217 | 0.88 | 39.88% |
| GS218 | 0.70 | 34.78% |
| GS219 | 0.78 | 36.51% |
| GS221 | 0.31 | 20.62% |
| GS224 | 0.67 | 34.11% |
| GS226 | 0.71 | 35.16% |
| GS228 | 0.69 | 34.21% |
| GS229 | 0.80 | 39.07% |
| GS230 | 0.63 | 31.31% |
| GS231 | 0.70 | 36.00% |
| GS232 | 0.67 | 33.12% |
| GS233 | 0.79 | 38.71% |
| GS234 | 0.79 | 39.77% |
| GS235 | 0.53 | 28.45% |
| GS236 | 0.82 | 39.93% |
| GS243 | 0.80 | 37.47% |
| GS244 | 0.74 | 36.13% |
| average | 0.50 | 29.54% |
