# Supplementary material for: A genomic perspective on the important genetic mechanisms of upland adaptation of rice
Source: BMC Plant Biol. 2014 Jun 11;14:160. doi: 10.1186/1471-2229-14-160 (PMC4074872; doi:10.1186/1471-2229-14-160)
Supplement: Additional file 10 — SNP annotation for the 166 accessions. a, using genome annotation information we classify the SNPs into intergenic SNPs and SNPs in gene region, which are further classified as SNPs in intron or UTR regions, non-synonymous SNPs and synonymous SNPs. b, SNPs causing radical mutations including disrupting stop/start codons, causing premature stop codons or disupting splicing sites. [file 1471-2229-14-160-S10.docx]

**b**

**a**

**Additional file 10** **SNP annotation for the 166 accessions.** a, using genome annotation information we classify the SNPs into intergenic SNPs and SNPs in gene region, which are further classified as SNPs in intron or UTR regions, non-synonymous SNPs and synonymous SNPs. b, SNPs causing radical mutations including disrupting stop/start codons, causing premature stop codons or disupting splicing sites.
