# Supplementary material for: A genomic perspective on the important genetic mechanisms of upland adaptation of rice
Source: BMC Plant Biol. 2014 Jun 11;14:160. doi: 10.1186/1471-2229-14-160 (PMC4074872; doi:10.1186/1471-2229-14-160)
Supplement: Additional file 11 — Wild rice information. [file 1471-2229-14-160-S11.docx]

| **Additional file 11** Wild rice information. | | | | |
| --- | --- | --- | --- | --- |
| Sample No. | Accession name | IRGC numbers | Origin | Population |
| rufipogon_105426 | URU WEE | 105426 | Sri Lanka | *rufipogon* |
| rufipogon_105912 | YALAMAN | 105912 | Thailand | *rufipogon* |
| rufipogon_105958 | PADI PADIAN | 105958 | Indonesia | *rufipogon* |
| rufipogon_105960 | null | 105960 | Bangladesh | *rufipogon* |
| rufipogon_106161 | null | 106161 | Laos | *rufipogon* |
| rufipogon_106505 | null | 106505 | Papua New Guinea | *rufipogon* |
| rufipogon_80506 | PERSAD/KAPNI | 80506 | India | *rufipogon* |
| rufipogon_81982 | null | 81982 | India | *rufipogon* |
| rufipogon_81991 | null | 81991 | Burma | *rufipogon* |
| rufipogon_Dongxiang | R_Dongxiang | Dongxiang | China | *rufipogon* |
| rufipogon_Nepal | R_Nepal | Nepal | Nepal | *rufipogon* |
| rufipogon_P25 | R_P25 |  | Guangdong, China | *rufipogon* |
| rufipogon_P46 | R_P46 |  | Hainan, China | *rufipogon* |
| rufipogon_P61 | R_P61 |  | Guangxi, China | *rufipogon* |
| rufipogon_YJ | R_YJ |  | Yunnan, China | *rufipogon* |
| nivara_103407 | null | 103407 | Sri Lanka | *nivara* |
| nivara_105327 | null | 105327 | India | *nivara* |
| nivara_105705 | null | 105705 | Nepal | *nivara* |
| nivara_105784 | null | 105784 | Thailand | *nivara* |
| nivara_105879 | JHORA DAN | 105879 | Bangladesh | *nivara* |
| nivara_106105 | null | 106105 | India | *nivara* |
| nivara_106154 | null | 106154 | Laos | *nivara* |
| nivara_106345 | null | 106345 | Burma | *nivara* |
| nivara_80470 | null | 80470 | India | *nivara* |
| nivara_89215 | null | 89215 | Cambodia | *nivara* |
