# Supplementary material for: A genomic perspective on the important genetic mechanisms of upland adaptation of rice
Source: BMC Plant Biol. 2014 Jun 11;14:160. doi: 10.1186/1471-2229-14-160 (PMC4074872; doi:10.1186/1471-2229-14-160)
Supplement: Additional file 12 — Phylogenetics analysis of the upland accessions. Green, black and orange branches respectively refer to upland, irrigated and wild accessions. The leaf nodes are labeled with the sample number of the accessions. This tree indicates that upland rice seems to have originated multiple times for the type Indica, while upland japonicas probably derived from a single origin. [file 1471-2229-14-160-S12.docx]

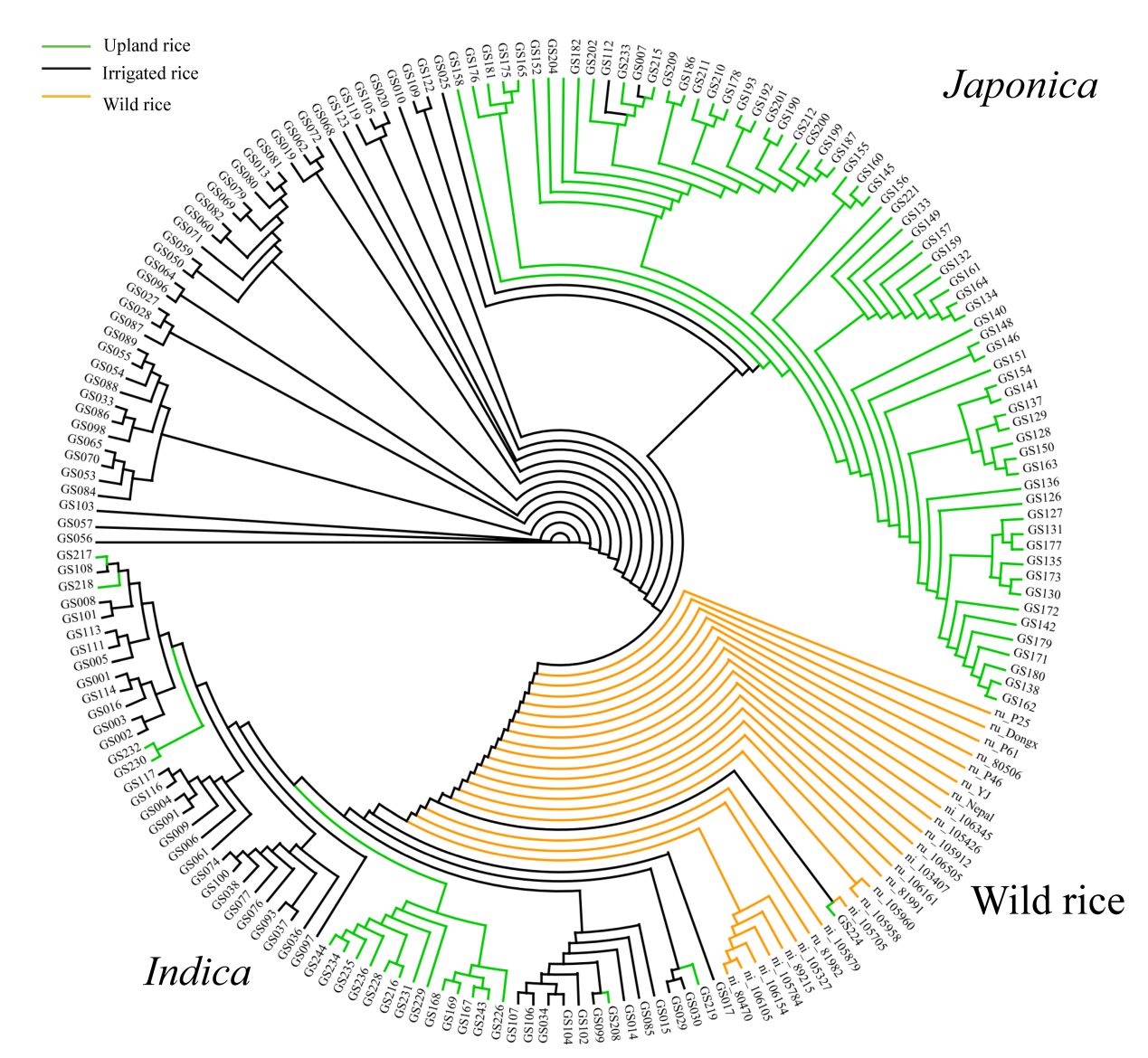


**Additional file 12 Phylogenetics analysis of the upland accessions.** Green, black and orange branches respectively refer to upland, irrigated and wild accessions. The leaf nodes are labeled with the sample number of the accessions (Additional file 2). This tree indicates that upland rice seems to have originated multiple times for the type indica, while upland japonicas probably derived from a single origin.
