# Supplementary material for: A genomic perspective on the important genetic mechanisms of upland adaptation of rice
Source: BMC Plant Biol. 2014 Jun 11;14:160. doi: 10.1186/1471-2229-14-160 (PMC4074872; doi:10.1186/1471-2229-14-160)
Supplement: Additional file 13 — Five of the six erroneously classified accessions. (a), typical indica seed; (b), typical japonica seed; (c) The accessions, GS190(TOS2300), GS192(WAB56-125), GS199(CNA4140), GS200 (GUARANI), GS201(Dourado), GS202(TGR78), were previously classified as upland indica according to their phenotypes (i.e. seed shape, etc.). According to our whole genome phylogenetics analysis, they are more close to upland japonica. GS200 was not photographed due to seeds being unavailable. [file 1471-2229-14-160-S13.docx]

**a b**


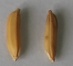

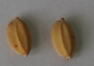


**c**

**
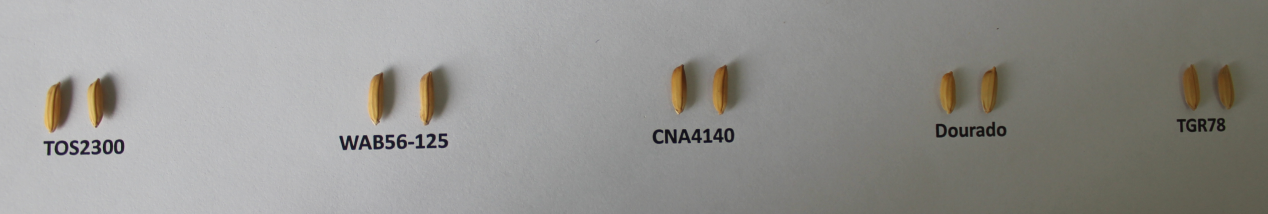
**

**Additional file 13 Five of the six erroneously classified accessions.** (a), typical indica seed; (b), typical japonica seed; (c) The accessions, GS190 (TOS2300), GS192(WAB56-125), GS199(CNA4140), GS200 (GUARANI), GS201(Dourado), GS202(TGR78), were previously classified as upland indica according to their phenotypes (i.e. seed shape, etc). According to our whole genome phylogenetics analysis, they are more close to upland japonica. GS200 was not photographed because its seeds were used up.
