# Supplementary material for: A genomic perspective on the important genetic mechanisms of upland adaptation of rice
Source: BMC Plant Biol. 2014 Jun 11;14:160. doi: 10.1186/1471-2229-14-160 (PMC4074872; doi:10.1186/1471-2229-14-160)
Supplement: Additional file 14 — EDR length distribution. Median length is 22721 bp. [file 1471-2229-14-160-S14.docx]

The EDR number

**Additional file 14 EDR length distribution.** Median length is 22721bp.
