# Supplementary material for: A genomic perspective on the important genetic mechanisms of upland adaptation of rice
Source: BMC Plant Biol. 2014 Jun 11;14:160. doi: 10.1186/1471-2229-14-160 (PMC4074872; doi:10.1186/1471-2229-14-160)
Supplement: Additional file 15 — Annotation of SNPs in EDRs. In EDRs, there are 8980 SNPs, of which 2409 are located within gene regions. In the gene regions, 241 and 266 SNPs are synonymous and non-synonymous, respectively. [file 1471-2229-14-160-S15.docx]

**Additional file 15** **Annotation of SNPs in EDRs.** In EDRs, there are 8980 SNPs, of which 2409 are located within gene regions. In the gene regions, 241 and 266 SNPs are synonymous and non-synonymous, respectively.
