# Supplementary material for: A genomic perspective on the important genetic mechanisms of upland adaptation of rice
Source: BMC Plant Biol. 2014 Jun 11;14:160. doi: 10.1186/1471-2229-14-160 (PMC4074872; doi:10.1186/1471-2229-14-160)
Supplement: Additional file 16 — Distribution pattern of SNPs in EDRs. [file 1471-2229-14-160-S16.docx]

| Additional file 16: Distribution pattern of SNPs in EDRs. | | |  |
| --- | --- | --- | --- |
| Genomic region | SNPs analyzed | SNPs in EDRs | Chi-square test  *P*-value |
| total SNPs | 3029822 | 8980 |  |
| intergenic regions | 2471373 | 6571 | 2.46E-11 |
| genic regions | 558449 | 2409 | < 2.2E-16 |
| UTR regions | 121640 | 392 | 0.1111 |
| coding sequences | 108907 | 503 | < 2.2E-16 |
| non-synonymous mutations | 61979 | 256 | 1.87E-07 |
| synonymous mutations | 47491 | 241 | < 2.2E-16 |
| Introns | 327339 | 1510 | < 2.2E-16 |

The SNPs in EDRs are significantly enriched in genic regions, both coding sequences and introns.
