# Supplementary material for: A genomic perspective on the important genetic mechanisms of upland adaptation of rice
Source: BMC Plant Biol. 2014 Jun 11;14:160. doi: 10.1186/1471-2229-14-160 (PMC4074872; doi:10.1186/1471-2229-14-160)
Supplement: Additional file 17 — Ecotype differentiated SNPs (EDS) between upland and irrigated populations. [file 1471-2229-14-160-S17.docx]

| Additional file 17: Ecotype differentiated SNPs (EDS) between upland and irrigated populations. | | | |
| --- | --- | --- | --- |
| Total EDS 2623 | Intergenic 2001 | Promoter 35 |  |
|  | Gene region 622 | UTR 92 |  |
|  |  | Coding sequences (CDS) 100 | 1 causes premature stop codon |
|  |  |  | 1 disrupts start codon |
|  |  |  | 55 non-synonymous |
|  |  | Intron 430 |  |

The EDS number in intergenic and genic regions (UTRs, coding sequences, introns).
