# Supplementary material for: A genomic perspective on the important genetic mechanisms of upland adaptation of rice
Source: BMC Plant Biol. 2014 Jun 11;14:160. doi: 10.1186/1471-2229-14-160 (PMC4074872; doi:10.1186/1471-2229-14-160)
Supplement: Additional file 18 — The 154 EDGs. [file 1471-2229-14-160-S18.docx]

| Additional file 18: 154 EDGs. | | |
| --- | --- | --- |
|  | Gene ID | Gene annotation |
| EDGs selected  in upland rice | *Os01g0646300* | Similar to RGA2 protein. |
|  | *Os01g0646400* | Non-protein coding transcript. |
|  | *Os01g0647200* | Hypothetical conserved gene. |
|  | *Os01g0651500* | Wax synthase domain containing protein. |
|  | *Os01g0646800* | Conserved hypothetical protein. |
|  | *Os03g0679300* | Similar to Shaggy-related protein kinase eta (EC 2.7.1.-) |
|  | *Os03g0368000* | Similar to Peroxidase 1. |
|  | *Os03g0368300* | Similar to Peroxidase 1. |
|  | *Os03g0368100* | Similar to Auxin responsive protein. |
|  | *Os03g0679100* | Vacuolar protein sorting-associated protein 26 family protein. |
|  | *Os03g0367900* | Peptidase C15, pyroglutamyl peptidase I family protein. |
|  | *Os04g0526300* | Sulfotransferase family protein. |
|  | *Os04g0526600* | Similar to Alpha-amylase/subtilisin inhibitor (RASI). |
|  | *Os04g0616600* | Serine/threonine protein kinase-related domain containing protein. |
|  | *Os04g0526101* | Non-protein coding gene. |
|  | *Os04g0526200* | Hypothetical conserved gene. |
|  | *Os04g0127200* | Similar to Subtilase. |
|  | *Os04g0526000* | Similar to Auxin-induced basic helix-loop-helix transcription factor. |
|  | *Os06g0541600* | Hypothetical conserved gene. |
|  | *Os07g0448400* | Similar to Plasma membrane integral protein ZmPIP2-6. |
|  | *Os09g0340500* | Hypothetical conserved gene. |
|  | *Os09g0385300* | Hypothetical protein. |
|  | *Os10g0527100* | Similar to Glutathione S-transferase, N-terminal domain containing protein. |
|  | *Os10g0527400* | Similar to Tau class GST protein 3. |
|  | *Os10g0364600* | Non-protein coding gene. |
|  | *Os10g0527601* | Glutathione S-transferase, C-terminal-like domain containing protein. |
|  | *Os10g0527800* | Similar to Tau class GST protein 3. |
|  | *Os10g0525800* | Similar to Glutathione S-transferase GSTU31 (Fragment). |
|  | *Os10g0364700* | Hypothetical protein. |
|  | *Os10g0527850* | Hypothetical gene. |
|  | *Os10g0527602* | Hypothetical gene. |
|  | *Os11g0486000* | Conserved hypothetical protein. |
|  | *Os11g0490200* | Protein kinase, core domain containing protein. |
|  | *Os11g0490300* | Hypothetical conserved gene. |
|  | *Os11g0490600* | Hypothetical conserved gene. |
|  | *Os12g0595800* | Similar to cDNA clone:J023098M23, full insert sequence. |
|  | *Os12g0597000* | Similar to Calcineurin B-like protein 2 (SOS3-like calcium binding protein 1). |
|  | *Os12g0529900* | Conserved hypothetical protein. |
|  | *Os12g0597400* | HI0933-like protein family protein. |
|  | *Os12g0530100* | Similar to Peroxidase 24 precursor (EC 1.11.1.7) (Atperox P24) (ATP47). |
|  | *Os12g0596850* | Non-protein coding gene. |
|  | *Os12g0530000* | Similar to Histone H2A. |
|  | *Os12g0597650* | Non-protein coding transcript. |
|  | *Os12g0597600* | K Homology, type 1, subgroup domain containing protein. |
|  | *Os12g0596200* | Similar to Zinc finger, C3HC4 type family protein, expressed. |
|  | *Os12g0596900* | Conserved hypothetical protein. |
|  | *Os12g0596000* | Similar to Lipoyltransferase (EC 2.3.1.-) (Lipoyl-[acyl-carrier protein]-protein- N-lipoyltransferase). |
|  | *Os12g0597100* | Similar to predicted protein. |
|  | *Os12g0597200* | Hypothetical protein. |
|  | *Os12g0598000* | Non-protein coding transcript. |
|  | *Os12g0597700* | Similar to WRKY DNA binding domain containing protein, expressed. |
|  | *Os12g0597500* | Peptidase M20 domain containing protein. |
|  | *Os12g0597300* | Similar to Mutator-like transposase-like protein. |
|  | *Os12g0596600* | Hypothetical protein. |
|  | *Os12g0596800* | Zinc finger, LIM-type domain containing protein. |
|  | *Os12g0596101* | Similar to predicted protein. |
|  | *Os12g0596300* | DC1 domain containing protein. |
|  | *Os12g0596100* | Similar to cDNA clone:J033047L20, full insert sequence. |
|  | *Os12g0597051* | Non-protein coding gene. |
|  | *Os12g0596501* | Hypothetical gene. |
| EDGs selected in irrigated rice | *Os01g0650100* | Hypothetical protein. |
|  | *Os01g0650000* | Similar to esterase. |
|  | *Os05g0369700* | Exo70 exocyst complex subunit family protein. |
|  | *Os05g0196600* | Similar to 1-aminocyclopropane-1-carboxylic acid synthase. |
|  | *Os05g0369500* | Exo70 exocyst complex subunit family protein. |
|  | *Os05g0196500* | Transcription factor jumonji-domain-containing protein. |
|  | *Os06g0711200* | Conserved hypothetical protein. |
|  | *Os06g0711100* | Conserved hypothetical protein. |
|  | *Os06g0187200* | Similar to Nucleotide sugar epimerase-like protein (UDP-D-glucuronate 4- epimerase) (EC 5.1.3.6). |
|  | *Os07g0655500* | Regulation of nuclear pre-mRNA protein domain containing protein. |
|  | *Os07g0451200* | Hypothetical conserved gene. |
|  | *Os07g0655600* | Pectin lyase fold/virulence factor domain containing protein. |
|  | *Os07g0655300* | Kelch related domain containing protein. |
|  | *Os07g0655400* | Protein of unknown function DUF1751, integral membrane, eukaryotic domain containing protein. |
|  | *Os07g0451101* | Hypothetical conserved gene. |
|  | *Os11g0481500* | Conserved hypothetical protein. |
|  | *Os11g0474600* | Terpenoid synthase domain containing protein. |
|  | *Os11g0481301* | Hypothetical gene. |
|  | *Os12g0255000* | Hypothetical gene. |
| EDGs selected in both populations | *Os01g0645650* | Similar to MYB transcription factor TaMYB1. |
|  | *Os01g0651932* | Hypothetical conserved gene. |
|  | *Os01g0643600* | Homeodomain-related domain containing protein. |
|  | *Os01g0645900* | Conserved hypothetical protein. |
|  | *Os01g0649900* | Lipase, GDSL domain containing protein. |
|  | *Os01g0651800* | Lipase, class 3 family protein. |
|  | *Os01g0643300* | Similar to PIN1-like auxin transport protein. |
|  | *Os01g0141000* | Similar to RAV-like protein. |
|  | *Os01g0645400* | Similar to Dimethylaniline monooxygenase-like protein (Flavin-containing monooxygenase YUCCA). |
|  | *Os01g0649200* | Lipase, GDSL domain containing protein. |
|  | *Os01g0646000* | Pentatricopeptide repeat domain containing protein. |
|  | *Os01g0652100* | Protein of unknown function DUF231, plant domain containing protein. |
|  | *Os01g0652000* | Conserved hypothetical protein. |
|  | *Os01g0649400* | Lipase, GDSL domain containing protein. |
|  | *Os01g0651866* | Hypothetical gene. |
|  | *Os01g0643666* | Non-protein coding gene. |
|  | *Os01g0643450* | Hypothetical gene. |
|  | *Os03g0680600* | Hypothetical conserved gene. |
|  | *Os03g0367800* | Zinc finger, HIT-type domain containing protein. |
|  | *Os03g0680400* | Hypothetical conserved gene. |
|  | *Os03g0680300* | Hypothetical conserved gene. |
|  | *Os03g0680200* | Hypothetical conserved gene. |
|  | *Os03g0680500* | Tetratricopeptide-like helical domain containing protein. |
|  | *Os04g0616500* | Hypothetical conserved gene. |
|  | *Os04g0316200* | Protein of unknown function DUF26 domain containing protein. |
|  | *Os04g0616400* | Similar to Receptor-like serine/threonine kinase. |
|  | *Os05g0192700* | Conserved hypothetical protein. |
|  | *Os06g0185100* | Similar to estradiol 17-beta-dehydrogenase 8. |
|  | *Os06g0186600* | Conserved hypothetical protein. |
|  | *Os06g0186000* | Conserved hypothetical protein. |
|  | *Os06g0185700* | Hypothetical conserved gene. |
|  | *Os06g0186400* | Similar to Serine carboxypeptidase II-2 precursor (EC 3.4.16.6) (CP-MII.2) (Fragment). |
|  | *Os06g0184600* | Hypothetical protein. |
|  | *Os06g0184733* | Hypothetical gene. |
|  | *Os06g0187000* | Non-protein coding transcript. |
|  | *Os06g0184700* | Hypothetical conserved gene. |
|  | *Os06g0185500* | Transferase family protein. |
|  | *Os06g0186700* | Biopterin transport-related protein BT1 family protein. |
|  | *Os06g0186100* | Hypothetical conserved gene. |
|  | *Os06g0184866* | Pentatricopeptide repeat domain containing protein. |
|  | *Os06g0184900* | Transferase family protein. |
|  | *Os06g0186900* | TIP49, C-terminal domain containing protein. |
|  | *Os06g0185800* | Pentatricopeptide repeat domain containing protein. |
|  | *Os06g0187100* | Similar to F26G16.17 protein. |
|  | *Os06g0185300* | Transferase family protein. |
|  | *Os06g0271300* | Similar to OSIGBa0140C02.7 protein. |
|  | *Os06g0185900* | Similar to Glutathione peroxidase. |
|  | *Os06g0271350* | Hypothetical gene. |
|  | *Os06g0187050* | Hypothetical gene. |
|  | *Os06g0185400* | Conserved hypothetical protein. |
|  | *Os06g0186500* | Similar to predicted protein. |
|  | *Os06g0184766* | Hypothetical conserved gene. |
|  | *Os06g0184800* | Similar to Low-temperature induced protein lt101.1 (Blt101) (Blt101.1). |
|  | *Os06g0186300* | Hypothetical conserved gene. |
|  | *Os07g0449100* | Similar to Light induced protein like. |
|  | *Os07g0450100* | Hypothetical conserved gene. |
|  | *Os07g0450000* | Similar to 60S ribosomal protein L44. |
|  | *Os07g0449700* | Similar to Type A response regulator 7. |
|  | *Os09g0340400* | Hypothetical protein. |
|  | *Os11g0474100* | Hypothetical protein. |
|  | *Os11g0474000* | Hypothetical protein. |
|  | *Os11g0485900* | Similar to NBS-LRR type resistance protein (Fragment). |
|  | *Os11g0474400* | Hypothetical protein. |
|  | *Os12g0529500* | Hypothetical conserved gene. |
|  | *Os12g0529300* | Similar to Auxin-binding protein (Fragment). |
|  | *Os12g0529400* | Similar to Auxin-binding protein 4 precursor (ABP). |
|  | *Os12g0529200* | Protein of unknown function DUF1618 domain containing protein. |
|  | *Os12g0240875* | TGF-beta receptor, type I/II extracellular region domain containing protein. |
|  | *Os12g0250900* | BRCT domain containing protein. |
|  | *Os12g0255200* | Staphylococcal nuclease (SNase-like), OB-fold domain containing protein. |
|  | *Os12g0240850* | Similar to POT family protein. |
| unassignable EDGs | *Os05g0131000* | Protein of unknown function DUF247, plant family protein. |
|  | *Os05g0131100* | Conserved hypothetical protein. |
|  | *Os06g0276300* | NB-ARC domain containing protein. |
|  | *Os11g0485500* | Conserved hypothetical protein. |

EDGs were assigned to four categories: EDGs specifically selected in upland ecotype, EDGs specifically selected in irrigated ecotype, EDGs selected in both ecotypes, and EDGs that cannot be assigned to specific ecotype.
