# Supplementary material for: A genomic perspective on the important genetic mechanisms of upland adaptation of rice
Source: BMC Plant Biol. 2014 Jun 11;14:160. doi: 10.1186/1471-2229-14-160 (PMC4074872; doi:10.1186/1471-2229-14-160)
Supplement: Additional file 19 — Enrichment analysis for interesting gene categories in EDGs and japonica-specific EDGs. [file 1471-2229-14-160-S19.docx]

Additional file 19: Enrichment analysis for interesting gene categories in EDGs and japonica-specific EDGs

| Gene categories | 2X2 contingency table | | | test | p value | interesting EDGs in this category |
| --- | --- | --- | --- | --- | --- | --- |
| lipase containing  GDSL |  | numbers in EDGs | numbers in whole genome gene list | chi square test | 7.49E-05 | Os01g0649900 Lipase, GDSL domain containing protein. |
|  | all gene cateogories | 154 | 42057 |  |  | Os01g0649200 Lipase, GDSL domain containing protein. |
|  | lipase containing GDSL | 3 | 80 |  |  | Os01g0649400 Lipase, GDSL domain containing protein. |
| peroxidase |  | numbers in EDGs | numbers in whole genome gene list | chi square test | 1.80E-03 | Os03g0368000 Similar to Peroxidase 1. |
|  | all gene cateogories | 154 | 42057 |  |  | Os03g0368300 Similar to Peroxidase 1. |
|  | peroxidase | 4 | 203 |  |  | Os06g0185900 Similar to Glutathione peroxidase. |
|  |  |  |  |  |  | Os12g0530100 Similar to Peroxidase 24 precursor. |
| Glutathione related |  | numbers in EDGs | numbers in whole genome gene list | chi square test | 5.44E-08 | Os06g0185900 Similar to Glutathione peroxidase. |
|  | all gene cateogories | 154 | 42057 |  |  | Os10g0527100 Similar to Glutathione S-transferase, N-terminal domain containing protein. |
|  | Glutathione related | 4 | 87 |  |  | Os10g0527601 Glutathione S-transferase, C-terminal-like domain containing protein. |
|  |  |  |  |  |  | Os10g0525800 Similar to Glutathione S-transferase GSTU31 (Fragment). |
| auxin signaling pathway |  |  |  | chi square test | 3.80E-06 | Os01g0643300 Similar to PIN1-like auxin transport protein. |
|  |  | numbers in EDGs | numbers in whole genome gene list |  |  | Os03g0368100 Similar to Auxin responsive protein. |
|  | all gene cateogories | 154 | 42057 |  |  | Os04g0526000 Similar to Auxin-induced basic helix-loop-helix transcription factor. |
|  | auxin signaling pathway | 5 | 178 |  |  | Os12g0529300 Similar to Auxin-binding protein (Fragment). |
|  |  |  |  |  |  | Os12g0529400 Similar to Auxin-binding protein 4 precursor (ABP) |
| mitogen-activated  protein kinase |  | numbers in Japonica-specific EDGs | numbers in whole genome gene list | chi square test | 1.37E-14 | Os01g0665200 Similar to Blast and wounding induced mitogen-activated protein kinase. |
|  | all gene cateogories | 122 | 42057 |  |  | Os01g0643800 Similar to Mitogen-activated protein kinase. |
|  | mitogen-activated protein kinase | 3 | 30 |  |  | Os08g0421800 Similar to Mitogen-activated protein kinase kinase kinase 1 |
| WRKY genes |  | numbers in Japonica-specific EDGs | numbers in whole genome gene list | chi square test | 0.03 | Os01g0665750 Similar to WRKY transcription factor 16. |
|  | all gene cateogories | 122 | 42057 |  |  | Os01g0665500 Similar to WRKY transcription factor 16. |
|  | mitogen-activated protein kinase | 2 | 101 |  |  |  |
| thioredoxin coding genes |  | numbers in Japonica-specific EDGs | numbers in whole genome gene list | chi square test | 0.04 | Os04g0367800 Thioredoxin, core domain containing protein. |
|  | all gene cateogories | 122 | 42057 |  |  | Os07g0657900 Similar to Thioredoxin reductase. |
|  | mitogen-activated protein kinase | 2 | 111 |  |  |  |
