# Supplementary material for: A genomic perspective on the important genetic mechanisms of upland adaptation of rice
Source: BMC Plant Biol. 2014 Jun 11;14:160. doi: 10.1186/1471-2229-14-160 (PMC4074872; doi:10.1186/1471-2229-14-160)
Supplement: Additional file 20 — Length distribution of selective sweep regions in the upland(a) and irrigated(b) population. Median length is 27002 bp(a) and 41001 bp(b) respectively. [file 1471-2229-14-160-S20.docx]

**a**

**b**

**Additional file 20 Length distribution of selective sweep regions in the upland(a) and irrigated(b) population.** Median length is 27002bp(a) and 41001bp(b) respectively.
