# Supplementary material for: A genomic perspective on the important genetic mechanisms of upland adaptation of rice
Source: BMC Plant Biol. 2014 Jun 11;14:160. doi: 10.1186/1471-2229-14-160 (PMC4074872; doi:10.1186/1471-2229-14-160)
Supplement: Additional file 21 — EDR scanning for type japonica. (a) FST values between upland japonica and irrigated japonica were plotted against physical coordinates over the genome; the red horizontal dashed line refers to the top 5‰ threshold. (b), XP-CLR scores were plotted against genomic coordinates; the green horizontal dashed line refers to the top 5‰ threshold. EDRs were obtained by taking the intersection of the EDRs given by the two approaches. The vertical dashed lines in (a) and (b) indicate the boundaries between two chromosomes. [file 1471-2229-14-160-S21.docx]

*F*_ST_


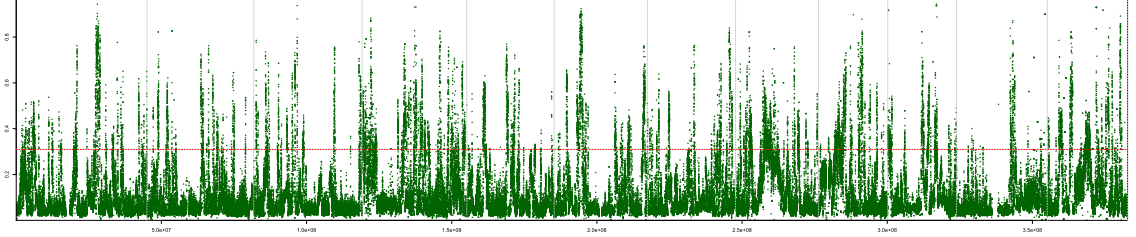


**a**


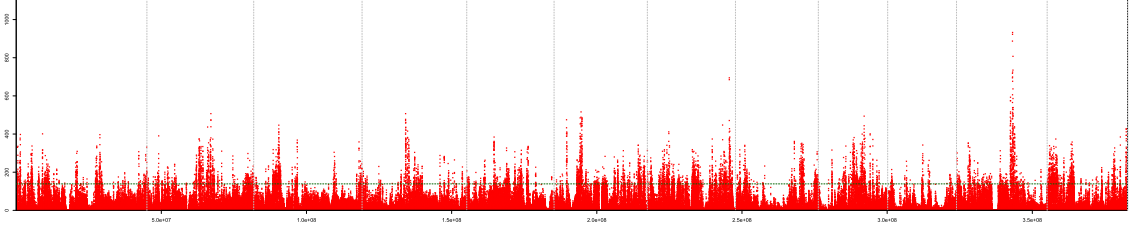


**b**

XP-CLR

**Additional file 21: EDR scanning for Japonica.** (a) *F*_ST_ values between upland japonica and irrigated japonica were plotted against physical coordinates over the genome; the red horizontal dashed line refers to the top 5‰ threshold. (b), XP-CLR scores were plotted against genomic coordinates; the green horizontal dashed line refers to the top 5‰ threshold. EDRs were obtained by taking the intersection of the EDRs given by the two approaches. The vertical dashed lines in (a) and (b) indicate the boundaries between two chromosomes.
