# Supplementary material for: A genomic perspective on the important genetic mechanisms of upland adaptation of rice
Source: BMC Plant Biol. 2014 Jun 11;14:160. doi: 10.1186/1471-2229-14-160 (PMC4074872; doi:10.1186/1471-2229-14-160)
Supplement: Additional file 22 — Length distribution of EDRs in type japonica. Median length is 24019 bp. [file 1471-2229-14-160-S22.docx]

The EDR number

**Additional file 22 Length distribution of EDRs in type japonica.** Median length is 24019bp.
