# Supplementary material for: A genomic perspective on the important genetic mechanisms of upland adaptation of rice
Source: BMC Plant Biol. 2014 Jun 11;14:160. doi: 10.1186/1471-2229-14-160 (PMC4074872; doi:10.1186/1471-2229-14-160)
Supplement: Additional file 23 — Detailed information of the 169 Japonica-specific EDGs. [file 1471-2229-14-160-S23.docx]

| Additional file 23: Detailed information of the 169 Japonica-specific EDGs. | |
| --- | --- |
| *Gene ID* | Gene annotation |
| *Os01g0645650* | Similar to MYB transcription factor TaMYB1. |
| *Os01g0665300* | NLI interacting factor domain containing protein. |
| *Os01g0646300* | Similar to RGA2 protein. |
| *Os01g0644500* | Hypothetical conserved gene. |
| *Os01g0663400* | Similar to aspartic proteinase oryzasin-1. |
| *Os01g0646400* | Non-protein coding transcript. |
| *Os01g0644400* | Hypothetical gene. |
| *Os01g0643600* | Homeodomain-related domain containing protein. |
| *Os01g0645900* | Conserved hypothetical protein. |
| *Os01g0665400* | Ribokinase family protein. |
| *Os01g0643900* | Oleosin family protein. |
| *Os01g0644000* | Twin-arginine translocation pathway signal domain containing protein. |
| *Os01g0534700* | Similar to MDR-like ABC transporter. |
| *Os01g0643300* | Similar to PIN1-like auxin transport protein. |
| *Os01g0645400* | Similar to Dimethylaniline monooxygenase-like protein (Flavin-containing monooxygenase YUCCA). |
| *Os01g0646000* | Pentatricopeptide repeat domain containing protein. |
| *Os01g0665750* | Similar to WRKY transcription factor 16. |
| *Os01g0665500* | Similar to WRKY transcription factor 16. |
| *Os01g0663500* | Transcriptional coactivator/pterin dehydratase family protein. |
| *Os01g0644200* | Conserved hypothetical protein. |
| *Os01g0665200* | Similar to Blast and wounding induced mitogen-activated protein kinase. |
| *Os01g0643800* | Similar to Mitogen-activated protein kinase. |
| *Os01g0643666* | Non-protein coding gene. |
| *Os01g0643450* | Hypothetical gene. |
| *Os02g0504000* | Cytochrome P450 domain containing protein. |
| *Os02g0173800* | Protein of unknown function DUF284, transmembrane eukaryotic family protein. |
| *Os02g0503850* | Similar to Cytochrome P450 CYP81L6. |
| *Os02g0173600* | Similar to Dreg-2 like protein. |
| *Os02g0173500* | Similar to Choline-phosphate cytidylyltransferase. |
| *Os02g0545000* | Similar to Myosin. |
| *Os02g0544951* | Similar to Unconventional myosin XI. |
| *Os02g0503900* | Cytochrome P450 family protein. |
| *Os02g0173700* | SAM (and some other nucleotide) binding motif domain containing protein. |
| *Os02g0504050* | Hypothetical conserved gene. |
| *Os03g0368000* | Similar to Peroxidase 1. |
| *Os03g0368300* | Similar to Peroxidase 1. |
| *Os03g0368100* | Similar to Auxin responsive protein. |
| *Os03g0680400* | Hypothetical conserved gene. |
| *Os03g0680300* | Hypothetical conserved gene. |
| *Os03g0841100* | Serine/threonine protein kinase-related domain containing protein. |
| *Os03g0841600* | UDP-glucuronosyl/UDP-glucosyltransferase family protein. |
| *Os03g0841700* | Similar to Prohibitin. |
| *Os03g0367900* | Peptidase C15, pyroglutamyl peptidase I family protein. |
| *Os03g0840900* | Protein of unknown function DUF543 family protein. |
| *Os03g0680500* | Tetratricopeptide-like helical domain containing protein. |
| *Os04g0526300* | Sulfotransferase family protein. |
| *Os04g0526600* | Similar to Alpha-amylase/subtilisin inhibitor (RASI). |
| *Os04g0527700* | CHCH domain containing protein. |
| *Os04g0526200* | Hypothetical conserved gene. |
| *Os04g0527800* | Mitochondrial substrate carrier family protein. |
| *Os04g0527900* | Similar to tonoplast membrane integral protein ZmTIP3-2. |
| *Os04g0527500* | Hypothetical protein. |
| *Os04g0368000* | Serine/threonine protein kinase-related domain containing protein. |
| *Os04g0528000* | Similar to OSIGBa0115K01-H0319F09.15 protein. |
| *Os04g0367800* | Thioredoxin, core domain containing protein. |
| *Os04g0527400* | BRO1 domain containing protein. |
| *Os04g0527950* | Hypothetical gene. |
| *Os05g0304100* | Conserved hypothetical protein. |
| *Os05g0304000* | Similar to Endo-1,4-beta-xylanase (Fragment). |
| *Os06g0275000* | Heading date 1. |
| *Os06g0274950* | Hypothetical gene. |
| *Os06g0277850* | Non-protein coding transcript. |
| *Os06g0276001* | Non-protein coding transcript. |
| *Os06g0273500* | Protein of unknown function DUF231, plant domain containing protein. |
| *Os06g0275900* | Forkhead-associated domain containing protein. |
| *Os06g0271600* | Zinc finger, RING/FYVE/PHD-type domain containing protein. |
| *Os06g0268800* | Uncharacterized protein family UPF0005 domain containing protein. |
| *Os06g0278450* | Hypothetical gene. |
| *Os06g0269300* | Six-bladed beta-propeller, TolB-like domain containing protein. |
| *Os06g0268600* | Non-protein coding transcript. |
| *Os06g0277600* | Conserved hypothetical protein. |
| *Os06g0274300* | Similar to Brassinosteroid insensitive 1- -associated receptor kinase 1. |
| *Os06g0271500* | Similar to OSIGBa0132O24.3 protein. |
| *Os06g0274000* | Similar to Heat shock factor binding protein 2. |
| *Os06g0275700* | Armadillo-like helical domain containing protein. |
| *Os06g0268900* | Non-protein coding transcript. |
| *Os06g0269000* | Hypothetical conserved gene. |
| *Os06g0277700* | Conserved hypothetical protein. |
| *Os06g0275500* | Similar to Polycomb protein EZ1 (Enhancer of zeste protein 1). |
| *Os06g0269200* | Protein of unknown function DUF6, transmembrane domain containing protein. |
| *Os06g0271300* | Similar to OSIGBa0140C02.7 protein. |
| *Os06g0276300* | NB-ARC domain containing protein. |
| *Os06g0274200* | Similar to H/ACA ribonucleoprotein complex subunit 2 (H/ACA snoRNP protein NHP2) |
| *Os06g0271350* | Hypothetical gene. |
| *Os06g0273800* | Similar to Signal peptidase 18 subunit (Fragment). |
| *Os06g0269100* | Hypothetical conserved gene. |
| *Os06g0275600* | Similar to TA1 protein (Fragment). |
| *Os06g0271400* | Hypothetical conserved gene. |
| *Os06g0274100* | Hypothetical conserved gene. |
| *Os06g0273400* | Conserved hypothetical protein. |
| *Os06g0267500* | Similar to NAM1 protein (Fragment). |
| *Os06g0278000* | Similar to carboxyl-terminal peptidase. |
| *Os06g0270200* | Similar to Potassium transporter 24. |
| *Os06g0275800* | Amino acid transporter, transmembrane domain containing protein. |
| *Os06g0271900* | Conserved hypothetical protein. |
| *Os06g0273700* | Protein of unknown function DUF231, plant domain containing protein. |
| *Os06g0268700* | Peptidase A1 domain containing protein. |
| *Os06g0272000* | Similar to Bacterial blight resistance protein. |
| *Os07g0661300* | Similar to Ubiquitin-specific protease 24. |
| *Os07g0449100* | Similar to Light induced protein like. |
| *Os07g0655500* | Regulation of nuclear pre-mRNA protein domain containing protein. |
| *Os07g0656100* | Similar to predicted protein. |
| *Os07g0661100* | Glycosyltransferase, family 4 protein. |
| *Os07g0655600* | Pectin lyase fold/virulence factor domain containing protein. |
| *Os07g0657300* | Conserved hypothetical protein. |
| *Os07g0656000* | Hypothetical conserved gene. |
| *Os07g0448100* | Similar to Plasma membrane integral protein ZmPIP2-6. |
| *Os07g0657600* | Hypothetical conserved gene. |
| *Os07g0657900* | Similar to Thioredoxin reductase. |
| *Os07g0655300* | Kelch related domain containing protein. |
| *Os07g0657500* | Zinc finger, C2H2-like domain containing protein. |
| *Os07g0658000* | Conserved hypothetical protein. |
| *Os07g0661201* | Non-protein coding gene. |
| *Os07g0658200* | Similar to 30S ribosomal protein S9. |
| *Os07g0448400* | Similar to Plasma membrane integral protein ZmPIP2-6. |
| *Os07g0657400* | Glycosyltransferase AER61, uncharacterized domain containing protein. |
| *Os07g0661400* | Conserved hypothetical protein. |
| *Os07g0655400* | Protein of unknown function DUF1751, integral membrane, eukaryotic domain containing protein. |
| *Os07g0658100* | Similar to predicted protein. |
| *Os07g0655800* | Similar to Aspartic acid-rich protein. |
| *Os07g0655900* | SNARE associated Golgi protein domain containing protein. |
| *Os07g0448200* | Similar to cDNA clone: J023132K03, full insert sequence. |
| *Os07g0661450* | Similar to predicted protein. |
| *Os07g0657950* | Hypothetical gene. |
| *Os07g0655850* | Hypothetical gene. |
| *Os07g0661500* | Hypothetical conserved gene. |
| *Os08g0421500* | Hypothetical conserved gene. |
| *Os08g0421800* | Similar to Mitogen-activated protein kinase kinase kinase 1 (EC 2.7.1.-). |
| *Os08g0421825* | Hypothetical gene. |
| *Os08g0421700* | Zinc finger, CCHC-type domain containing protein. |
| *Os08g0421400* | Conserved hypothetical protein. |
| *Os08g0421850* | U-box domain containing protein. |
| *Os09g0407800* | Pentatricopeptide repeat domain containing protein. |
| *Os09g0395600* | Similar to Xyloglucan endotransglucosylase/hydrolase 13. |
| *Os09g0409000* | Conserved hypothetical protein. |
| *Os09g0407600* | Similar to DNA mismatch repair protein. |
| *Os09g0407700* | Haloacid dehalogenase-like hydrolase domain containing protein. |
| *Os09g0395300* | Similar to myb-like DNA-binding domain, SHAQKYF class family protein. |
| *Os09g0407900* | Peptidase C19, ubiquitin carboxyl-terminal hydrolase 2 family protein. |
| *Os09g0407950* | Similar to transducin family protein / WD-40 repeat family protein. |
| *Os09g0410500* | Similar to teosinte-branched one. |
| *Os09g0410400* | Zinc finger, RING-type domain containing protein. |
| *Os09g0408000* | Tetratricopeptide-like helical domain containing protein. |
| *Os09g0395400* | Conserved hypothetical protein. |
| *Os09g0408900* | Similar to predicted protein. |
| *Os11g0474100* | Hypothetical protein. |
| *Os11g0486000* | Conserved hypothetical protein. |
| *Os11g0489600* | ATPase, P-type, plasma-membrane proton-efflux domain containing protein. |
| *Os11g0474000* | Hypothetical protein. |
| *Os11g0485900* | Similar to NBS-LRR type resistance protein (Fragment). |
| *Os11g0474400* | Hypothetical protein. |
| *Os11g0474500* | Conserved hypothetical protein. |
| *Os11g0474450* | Hypothetical gene. |
| *Os11g0485200* | ATPase, P-type, K/Mg/Cd/Cu/Zn/Na/Ca/Na/H-transporter family protein. |
| *Os11g0485000* | Conserved hypothetical protein. |
| *Os12g0597000* | Similar to Calcineurin B-like protein 2 (SOS3-like calcium binding protein 1). |
| *Os12g0597400* | HI0933-like protein family protein. |
| *Os12g0596850* | Non-protein coding gene. |
| *Os12g0597600* | K Homology, type 1, subgroup domain containing protein. |
| *Os12g0596900* | Conserved hypothetical protein. |
| *Os12g0597100* | Similar to predicted protein. |
| *Os12g0597200* | Hypothetical protein. |
| *Os12g0598000* | Non-protein coding transcript. |
| *Os12g0248600* | Hypothetical protein. |
| *Os12g0597500* | Peptidase M20 domain containing protein. |
| *Os12g0597300* | Similar to Mutator-like transposase-like protein. |
| *Os12g0596600* | Hypothetical protein. |
| *Os12g0596800* | Zinc finger, LIM-type domain containing protein. |
| *Os12g0597051* | Non-protein coding gene. |
