# Supplementary material for: A genomic perspective on the important genetic mechanisms of upland adaptation of rice
Source: BMC Plant Biol. 2014 Jun 11;14:160. doi: 10.1186/1471-2229-14-160 (PMC4074872; doi:10.1186/1471-2229-14-160)
Supplement: Additional file 24 — Detailed information of the 56 Indica-specific EDGs. [file 1471-2229-14-160-S24.docx]

Additional file 24: Detailed information of the 56 Indica-specific EDGs.

| Os01g0552300 | Similar to Protein phosphatase-2C. |
| --- | --- |
| Os01g0552100 | Non-protein coding transcript. |
| Os01g0547200 | Zinc finger, RING/FYVE/PHD-type domain containing protein. |
| Os01g0549850 | Non-protein coding gene. |
| Os01g0550000 | DEAD-like helicase, N-terminal domain containing protein. |
| Os01g0572700 | EF hand domain containing protein. |
| Os01g0547133 | Protein of unknown function DUF239, plant domain containing protein. |
| Os01g0549700 | Similar to cDNA clone:J033114G10, full insert sequence. |
| Os03g0713700 | Hypothetical conserved gene. |
| Os03g0724100 | Phosphatidylinositol transfer protein-like, N-terminal domain containing protein. |
| Os03g0714100 | Conserved hypothetical protein. |
| Os03g0719900 | Similar to Peptide transporter 1. |
| Os03g0713100 | Similar to dynamin-related protein 1C. |
| Os03g0713400 | Similar to NADH-ubiquinone oxidoreductase 75 kDa subunit |
| Os03g0723400 | Conserved hypothetical protein. |
| Os03g0717600 | Zinc finger, C2H2-type matrin domain containing protein. |
| Os03g0723700 | Conserved hypothetical protein. |
| Os03g0723800 | Conserved hypothetical protein. |
| Os03g0724500 | Conserved hypothetical protein. |
| Os03g0723600 | Conserved hypothetical protein. |
| Os03g0722800 | Cyclin-like F-box domain containing protein. |
| Os03g0715600 | Similar to EF hand family protein. |
| Os03g0713200 | Conserved hypothetical protein. |
| Os03g0714400 | Similar to ATP binding protein. |
| Os03g0715400 | Membrane protein,Tapt1/CMV receptor domain containing protein. |
| Os03g0724300 | Tyrosine protein kinase domain containing protein. |
| Os03g0717200 | Cytochrome b561 family protein. |
| Os03g0723250 | Non-protein coding gene. |
| Os03g0713500 | Similar to Mitogen-activated protein kinase kinase 10-2. |
| Os03g0722600 | Adenylate cyclase-associated CAP domain containing protein. |
| Os03g0722700 | Similar to SWI2/SNF2-like protein (Fragment). |
| Os03g0715500 | Armadillo-type fold domain containing protein. |
| Os03g0713000 | Similar to Chloroplast threonine deaminase 1. |
| Os03g0717000 | Similar to TMK protein precursor. |
| Os03g0720300 | Similar to Glutamate decarboxylase. |
| Os03g0713600 | Aldo/keto reductase domain containing protein. |
| Os03g0712900 | Conserved hypothetical protein. |
| Os03g0723000 | GRAS transcription factor domain containing protein. |
| Os03g0715332 | Hypothetical conserved gene. |
| Os07g0206600 | Similar to Hexose transporter. |
| Os07g0205000 | Similar to Ubiquinol-cytochrome c reductase complex 14 kDa protein. |
| Os07g0206300 | Hypothetical protein. |
| Os07g0206900 | Protein of unknown function DUF642 family protein. |
| Os07g0204900 | Similar to Zeta-carotene desaturase. |
| Os07g0206700 | Similar to Cycloartenol-C-24-methyltransferase 1. |
| Os07g0204800 | Similar to Charged multivesicular body protein 2a. |
| Os07g0206800 | Hypothetical conserved gene. |
| Os07g0206400 | 13 kDa prolamin precursor. |
| Os07g0207100 | Protein kinase, core domain containing protein. |
| Os07g0206750 | Hypothetical gene. |
| Os07g0206500 | 13 kDa prolamin precursor. |
| Os07g0206650 | Hypothetical gene. |
| Os07g0206850 | Hypothetical gene. |
| Os09g0416500 | Hypothetical conserved gene. |
| Os09g0416200 | Similar to Glucose transporter (Fragment). |
| Os09g0416250 | Hypothetical gene. |
