# Supplementary material for: A genomic perspective on the important genetic mechanisms of upland adaptation of rice
Source: BMC Plant Biol. 2014 Jun 11;14:160. doi: 10.1186/1471-2229-14-160 (PMC4074872; doi:10.1186/1471-2229-14-160)
Supplement: Additional file 26 — Nine genes from the indica XP-CLR top signals overlap with the previous EDGs of merged population. [file 1471-2229-14-160-S26.docx]

Additional file 26: nine genes from the indica XP-CLR top signals overlap with the previous EDGs of merged population

| Os06g0185100 | Similar to estradiol 17-beta-dehydrogenase 8. |
| --- | --- |
| Os06g0184733 | Hypothetical gene. |
| Os06g0184700 | Hypothetical conserved gene. |
| Os06g0184866 | Pentatricopeptide repeat domain containing protein. |
| Os06g0184900 | Transferase family protein. |
| Os06g0185300 | Transferase family protein. |
| Os06g0185400 | Conserved hypothetical protein. |
| Os06g0184766 | Hypothetical conserved gene. |
| Os06g0184800 | Similar to Low-temperature induced protein lt101.1 |
