# Supplementary material for: A genomic perspective on the important genetic mechanisms of upland adaptation of rice
Source: BMC Plant Biol. 2014 Jun 11;14:160. doi: 10.1186/1471-2229-14-160 (PMC4074872; doi:10.1186/1471-2229-14-160)
Supplement: Additional file 27 — Four genes from the indica XP-CLR top signals overlap with the previous EDGs of the japonica population. [file 1471-2229-14-160-S27.docx]

Additional file 27: four genes from the indica XP-CLR top signals overlap with the previous EDGs of the japonica population

| Os06g0277600 | Conserved hypothetical protein. |
| --- | --- |
| Os06g0274300 | Similar to BRASSINOSTEROID INSENSITIVE 1-associated receptor kinase 1. |
| Os06g0277700 | Conserved hypothetical protein. |
| Os06g0274200 | Similar to H/ACA ribonucleoprotein complex subunit 2 |
